# A distributed subcortical circuit linked to instrumental information-seeking about threat

**DOI:** 10.1073/pnas.2410955121

**Published:** 2025-01-15

**Authors:** Hailey A. Trier, Nima Khalighinejad, Sorcha Hamilton, Caroline Harbison, Luke Priestley, Mark Laubach, Miriam Klein-Flügge, Jacqueline Scholl, Matthew F. S. Rushworth

**Affiliations:** ^a^Wellcome Centre for Integrative Neuroimaging, Department of Experimental Psychology, University of Oxford, Oxford OX1 3TA, United Kingdom; ^b^Department of Neuroscience, American University, Washington, DC 20016; ^c^Department of Psychiatry, University of Oxford, Warneford Hospital, Oxford OX3 7JX, United Kingdom; ^d^Université Claude Bernard Lyon 1, CNRS, INSERM, Lyon Neuroscience Research Center U1028 UMR5292, PsyR2 Team, Centre Hospitalier Le Vinatier, 9678 Bron, France; ^e^Wellcome Centre for Integrative Neuroimaging, Centre for Functional MRI of the Brain, University of Oxford, Nuffield Department of Clinical Neurosciences, John Radcliffe Hospital, Oxford OX3 9DU, United Kingdom

**Keywords:** decision-making, information seeking, cognitive neuroscience, threat, reward

## Abstract

Daily life for humans and other animals requires switching between periods of threat- and reward-oriented behavior. We investigated neural activity associated with spontaneous switching, in a naturalistic task, between foraging for rewards and seeking information about potential threats with 7T fMRI in healthy humans. Switching was driven by estimates of likelihood of threat and reward. Both tracking of threat and switching to a vigilant mode in which people sought more information about potential threats were associated with specific but distributed patterns of activity spanning habenula, dorsal raphe nucleus (DRN), anterior cingulate cortex, and anterior insula cortex. Different aspects of the distributed activity patterns were linked to monitoring the threat level, seeking information about the threat, and actual threat detection. A distinct pattern of activity in the same circuit and elsewhere occurred during returns to reward-oriented behavior. Individual variation in DRN activity reflected individual variation in the seeking of information about threats.

## Significance

This study a) records activity in the human dorsal raphe nucleus (DRN); b) links it to a specific aspect of behavior; and c) sets out an account of how the activity arises through interactions across a distributed neural circuit (critically comprising habenula and insula). The behavior we investigate is quotidian and intuitively understandable but has received little scholarly attention, and the behavior we link to DRN is the same as one that we have shown in large-scale studies to be strongly and robustly related to individual variation in anxiety.

For most animals, daily life entails careful balancing between foraging for food while maintaining vigilance for predatory threats. In many cases, animals alternate between periods when behavior is oriented toward foraging for food and seeking information about potential threats. This is a ubiquitous feature of behavior that can be observed in many animals, not only in the wild but in suburban parks and gardens. For example, squirrels alternate between focusing on their search for food and looking around to check for predators. Alternating between reward-focused behavior and threat checking is equally a core feature of daily life for modern humans; we are similarly drawn away from projects we wish to pursue (work or leisure plans) by intermittent periods of anxiety and focus on potential threats. Despite considerable interest in the brain mechanisms linked to many aspects of reward, curiosity, and threat, little is known about how humans or other animals switch between reward-oriented behavior and threat-oriented information seeking (1, 2). We, therefore, devised a task that allowed investigation of self-initiated, uncued switches in behavioral focus between reward pursuit and checking for threats in humans. We employed a naturalistic, ethologically inspired task designed to tap into the foraging and survival problems that humans and other animals have evolved to solve (3–5).

We also used ultra-high field functional MRI (7T fMRI) to identify distributed patterns of brain activity that accompanied transitions between reward orientation and threat orientation. The ubiquity of the need to balance reward-guided foraging and threat-oriented information seeking across animals suggests an evolutionarily ancient origin. Some of the first cephalic neural circuits to have evolved remain present in the human brain; however, they also remain comparatively small in size. As a result, they are often overlooked, and difficult to measure using conventional neuroimaging methods. Here, however, by using high resolution (1.5 mm isotropic), rapid repetition time (1.96 s), accelerated ultra-high field (7T) imaging we focused on one such candidate neural circuit centered on the habenula (Hb) and dorsal raphe nucleus (DRN). Importantly, we used a rigorous filtering approach to exclude potential artifacts from BOLD data by, for example, measuring heart rate and respiration to regress out the effect of physiological noise which is necessary for accurate brainstem imaging (see *Methods* for details).

While the Hb–DRN circuit is present in primates it is unusual in that it is present in many vertebrates including even cyclostomes—jawless fish—that diverged from other vertebrates 550 mya (6, 7). The DRN is an important source of serotonergic innervation and like other neuromodulatory systems, such as the dopaminergic system with its origins in the ventral tegmental area (VTA) and substantia nigra pars compacta (SN), its activity is under Hb control. We have focused on these nuclei in previous investigations of action initiation in reward-guided contexts (8–11). While activity in VTA and especially SN has been linked to action initiation, activity in DRN tracks changes in the environment such as average reward rate (8–11). Activity in Hb reflects aspects of choice value but has also been linked to action inhibition and disinhibition and initiation (7, 12, 13). The present experimental design allows us to test whether, in addition to reward rate, DRN also tracks other relatively slowly varying features of the environment such as estimates of threat level. It also makes it possible to investigate whether activity in DRN and/or Hb, VTA, and SN might be related to changes in behavioral orientation—switching from foraging to checking for threat or vice versa—even if its activity is not closely linked to action initiation per se. Factors such as reward rate and threat level should determine how advantageous it is to forage and disadvantageous it is not to check for threats. Our interest, therefore, was in examining whether DRN, VTA, SN, or Hb tracked threat level and/or reward rate and the process of switching between behaviors: foraging and checking for threat.

Other considerations suggest that each of these four areas might be linked to threat-oriented information seeking. DRN is part of the serotonergic system which is a first line pharmacological target in depression and anxiety (14). However, comparatively little is known about how human DRN activity is linked to behavior in the healthy brain. Even though our current study is conducted with a sample of healthy human participants, a previous large scale, on-line behavioral study (15) has established the relevance of this task for examining behavior linked to individual differences in psychiatric dimensions including compulsive checking and apathy. In monkeys, Hb and potentially dopaminergic areas such as VTA and SN, have been linked to information seeking albeit in contexts in which the animals are seeking information about possible future rewards (1). The degree to which a specific neural structure is involved in information seeking can vary dramatically depending on the type of information seeking (16). Whether these areas’ activity is also linked to seeking information about potential threats can be investigated in the current experiment.

In the mammalian brain Hb–DRN interactions are likely to be influenced by cortical activity but little is known about how this occurs. There is little direct information about Hb connections in primates but only two cortical areas, anterior cingulate cortex (ACC) and anterior insula (AI), are known to project to Hb in rodents (17). DRN projects widely in the cortex but the same two areas, ACC and AI, have some of the strongest projections to DRN (18). ACC, but not AI, projections have been investigated in primates and projections to both Hb and DRN have been reported. Moreover, in macaques, albeit in other contexts, ACC and AI carry signals related to those in DRN (8, 11) and ACC has been linked to information seeking (1, 19–21). We therefore also report activity from ACC and AI. We use a similar approach to that employed in previous studies in which we considered activity in predefined regions of interest (ROIs) in the subcortical regions Hb, DRN, VTA, and SN (8–11). Then, as previously, we use analysis of activity across the whole brain to identify regions of cortical activity linked to the task.

## Results

**Fig. 1.**
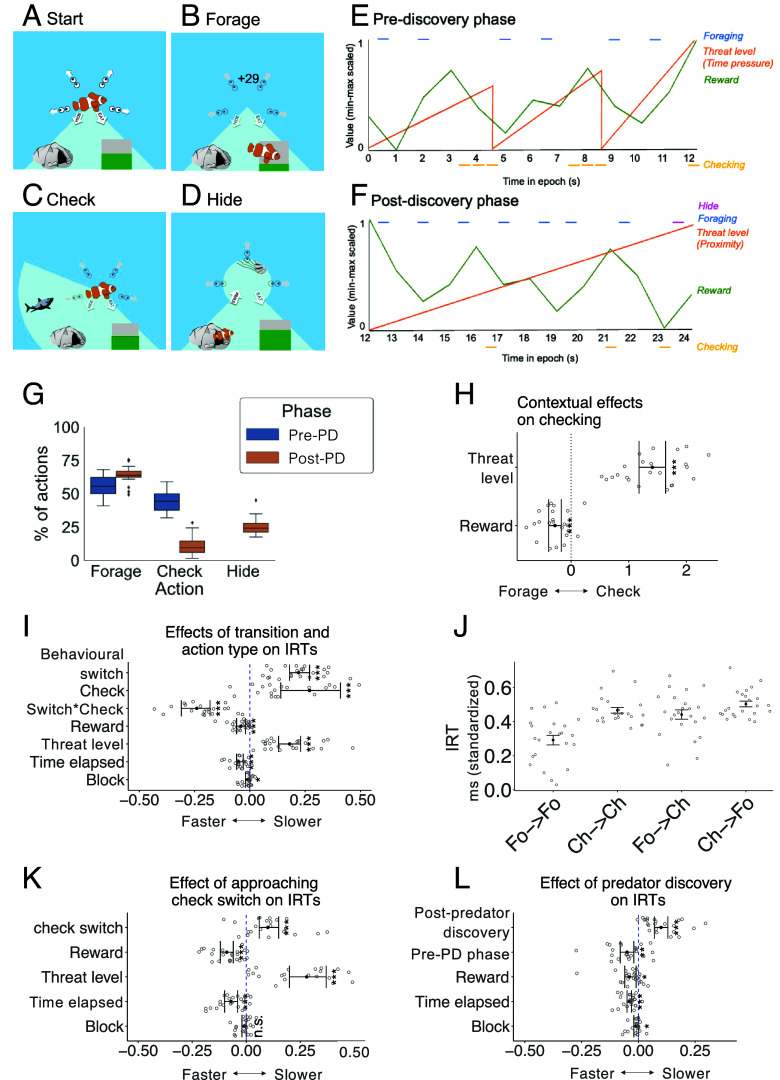
Experimental task. (*A*) At the start of a block, the fish begins in the center position. In this block, the surrounding area is divided into four sections that must be searched for predators. At left (not shown here) participants see their collected energy, number of lives gained (each "life" = 1 bar of energy), and time remaining in the current block (gray/black wheel). (*B*) When foraging the fish dives down into the patch of food and the energy bar increases by the amount of reward gained. (*C*) When checking, the fish checks in a particular direction. In this example, the fish discovers that there is a predator in the area being checked. (*D*) When hiding, the fish dives into a cave and is safe from the predator. It can see the predator reach the screen center and then retreat, and with another button press the fish returns to the original center position. The task lacked a traditional trial structure and participants freely chose when to forage or check. (*E*) Threat level and reward rate varied during the course of each block both before and (*F*) after predator discovery (pre-PD and post-PD. In the pre-PD phase, we used the time that had elapsed since the last check (or the start of the block) as a measure of time pressure and as the participants’ estimate of the imminence of threat. In the post-PD phase, participants now had more information about the predator because they had, by definition, observed its actual proximity to their fish avatar. We therefore used proximity as the index of threat imminence in the post-PD phase. (*G*) Box plots showing action type as a percentage of all actions in each phase. (*H*) Group mean estimates and 95% CI from two-tailed single sample *t* tests showing effects of model parameters on the probability that an action was a check as opposed to a forage. (*I*–*L*) Group mean estimates and 95% CI from two-tailed single sample *t* tests showing effects of model parameters on interresponse times (IRTs). (*I*) There were main effects of behavioral switch, checking, and time pressure on IRTs indicating that each significantly slowed IRTs (all *P* < 0.001). IRTs were significantly faster during higher rewards (*P* < 0.001). There was also a significant interaction between behavioral switch and checking (*P* < 0.001). (*J*) Group mean and SE for the average IRTs (standardized) associated with each action sequence within each participant. IRTs were slowest when switching from checking to forage and fastest when repeating foraging. (*K*) IRTs between forages became significantly slower as they approached an upcoming switch to checking (*P* < 0.001). (*L*) There was a significant main effect of whether an action occurred immediately after discovering a new predator; discovering a predator slowed IRTs (*P* < 0.001; both pre- and post-PD phase data analyzed). *P < 0.05, **P < 0.01, ***P < 0.001, n.s. = not significant.

We employed a gamified and continuous, but carefully controlled, task in which both reward and threat stimuli were varied independently across the experiment (Fig. 1). We measured behavior when it was predominantly guided by reward (foraging) and when it was predominantly guided by threat (checking). In both behavioral contexts, we varied two environmental features that might be expected to influence switching between the two behaviors: rate of reward and an estimate of threat level (estimated time until a predator could reach the participant) that we refer to as time pressure. Importantly, participants chose themselves when and how frequently to switch from one behavior to the other. In other words, the task allows participants to decide when to engage in a threat-related response and when to pursue reward without being externally cued.

Participants were trained on the task in an online session before the scan. During the task (Fig. 1 *A*–*D*) participants used arrow keys to control an animated fish in an ocean environment. The environment contained several important features. First, there was rewarding food, later translated to a bonus payment. By varying their rate, we manipulated the reward rate parameter. Second, there were threatening predators which caused “virtual death” if they “caught” the fish. Virtual death was signaled visually and led to the end of the epoch and a loss of 100 points which participants found aversive and generally avoided. There were three different predator types and participants learned that they approached at different speeds. The range assisted in the manipulation of threat level (time pressure). Third, there was a hiding space where participants could hide from the predators and avoid being caught. Thus, overall, participants attempted to gain as much reward as possible while avoiding being caught by a predator.

In the absence of a traditional task trial structure, human participants freely and continuously chose between foraging for reward or checking for predatory threat in the virtual environment during 27 blocks each lasting 90 s. Here, we focus on the two most frequent actions: foraging and checking for threat, and the two environmental variables that principally motivated each of the behaviors: reward rate and time pressure—an index of threat level. A third action, hiding, was also available and was taken on occasions when the threat was imminent. Pressing the “hide” button caused the fish to escape to a safe space where it could not be caught by the predator; a subsequent button press returned the fish to the center (Fig. 1*D*). Because hiding only occurred once per block, there are insufficient data for a full analysis of hiding-related behavior and brain activity.

Reward rate corresponded to the average amount of food available and followed a random walk (range: 0 to 90 units). Participants could always see how much food was available [proportion of green vs. gray bar on lower right of display (Fig. 1 *A*–*D*)]. When participants took the foraging action, the fish dived down to obtain food. The index of threat—time pressure—corresponded to the time elapsed after the start of the block and multiplied by the speed of the predator (the participant was informed of this speed and had experienced it previously during practice blocks performed prior to scanning). As time elapsed, the predator was more likely to get closer to the center of the screen and, therefore, more likely to catch the participant’s fish avatar (*Methods* and *SI Appendix*, Eq. **S1**). In the post-PD phase, participants gained more information about the predator because they had seen its actual position and so the estimate of threat level was adjusted appropriately (proximity measure: *Methods* and *SI Appendix*, Eqs. **S2** and **S3**). Importantly, the reward rate at any moment was decorrelated from time pressure so that the brain activity related to each variable could be separated and identified (*SI Appendix*, Fig. S1 illustrates the lack of collinearity between these task variables and others).

Predators were hidden from participants’ view unless participants pressed a button to “check” a portion of the surrounding area (Fig. 1*C*). At that point they were able to see a segment of the environment in which there might be a predator. Predators appeared (after a random delay, 2 to 10.5 s) at the edge of the screen and moved toward the fish’s location at the screen center. When the predator reached the screen center it either caught the fish, ending the epoch, or, if the fish was in hiding, the predator simply exited the environment (ending the epoch).

Actions entailed time costs, so participants had to manage their time strategically to maximize reward. For example, each foraging action took 1.5 s (*Timings*). After making one foraging action, participants had to make another to obtain further reward. Alternatively, after the 1.5 s time elapsed, participants could switch to checking or hiding.

### Participants Used Task-Relevant Information to Guide Behavior.

**Fig. 2.**
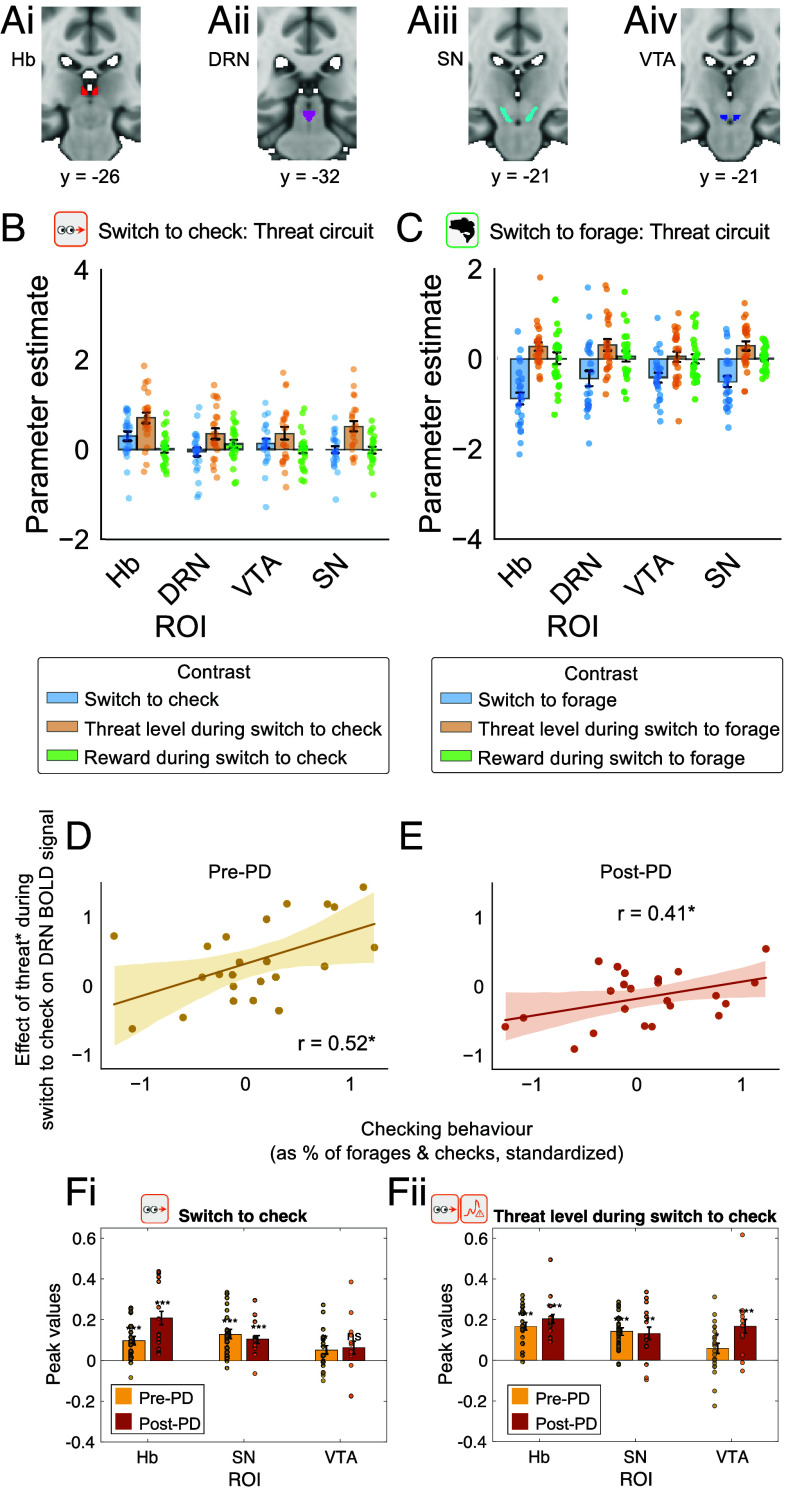
Time pressure and switches to checking are associated with activity increases in subcortical areas. (*A*) Activity in all four subcortical ROIs—Hb (*A*-i), DRN (*A*-ii), SN (*A*-iii), and VTA (*A*-iv)—is shown during switches to checking (*B*) and foraging (*C*). Activity in all areas was correlated with time pressure (an index of threat level), increased during switches to checking, decreased during switches to foraging, but carried little information about reward both during checking and foraging. All results are from analyses of pre-PD phase. (*D*) BOLD signal in DRN corresponding to threat level during switching to checking and switching to checking was significantly related to checking behavior as measured by checks as a percentage of forages and checks combined in the pre-PD phase (*E*) and in the post-PD phase. (*F*) The check (*Fi*) and threat level (*Fii*; time pressure) effects in the other areas—Hb, VTA, and SN—were similar in the pre-PD (yellow) and post-PD (orange) phases.

**Fig. 3.**
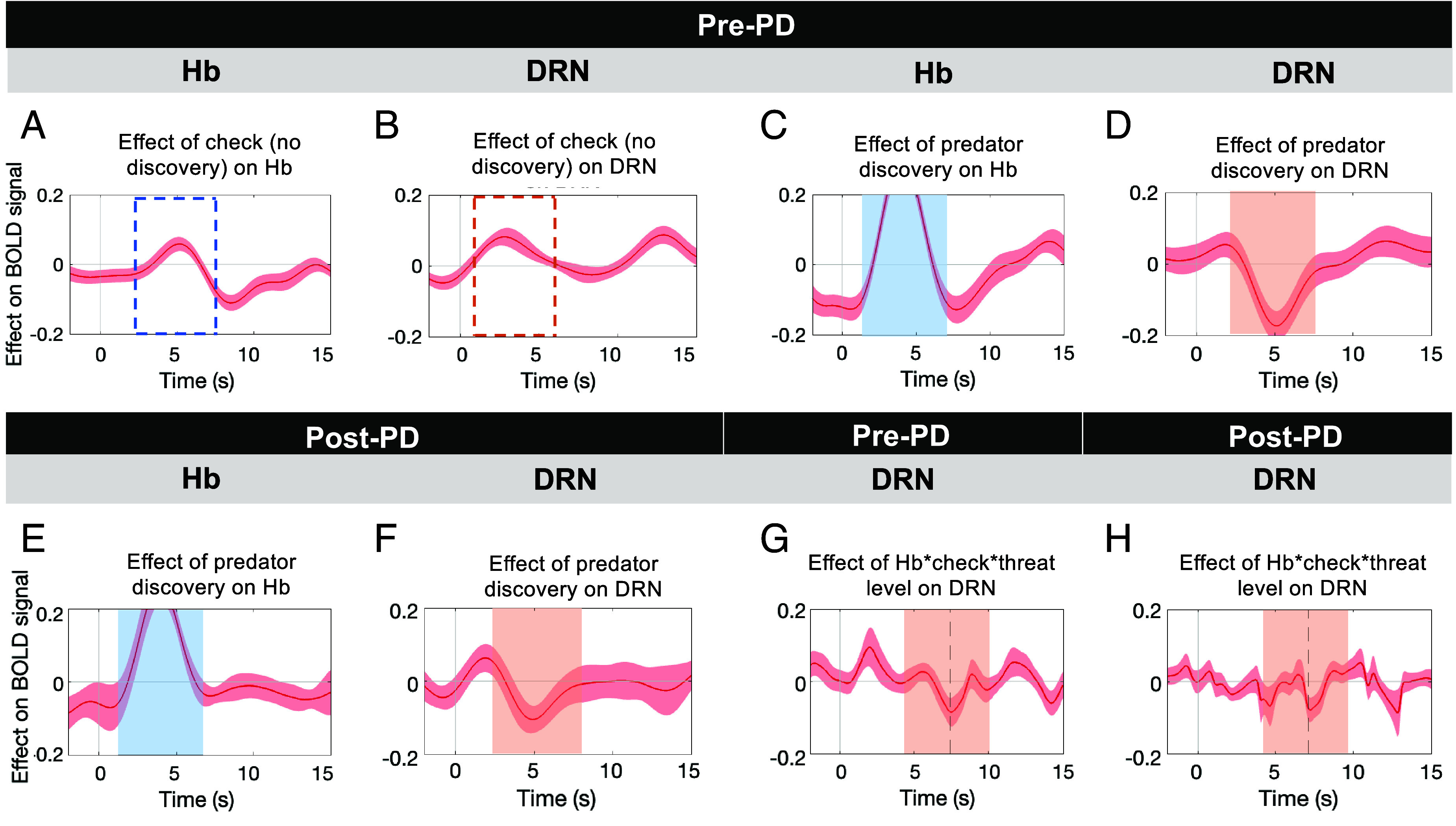
Activity in Hb and DRN during predator discovery vs. nondiscovery. Activity in Hb (*A*; dotted blue area) and DRN (*B*; dotted ochre area) initially increased when participants switched to checking during the pre-PD phase and no predator was detected. The increase began earlier in DRN but overlapped in time. When checking led to predator discovery, however, the initial period of increase was more marked in Hb (*C*, area corresponding to blue box in panel *A*) and it was followed by a marked period of inhibition of DRN activity (*D*; area corresponding to ochre area in panel *B*). The same patterns, albeit slightly diminished in size, were found again in Hb (*E*), DRN (*F*) in the post-PD phase when checking also led to predator detection. When we examined Hb–DRN interactions as a function of all the factors shown to influence both Hb and DRN—checking (vs. foraging), detecting the predator, and the threat level—we observed periods of strong negative interactions in both pre-PD (*G*) and post-PD (*H*) phases. These occurred just at the end of the period of negative DRN activity change in panels *D* and *F*.

**Fig. 4.**
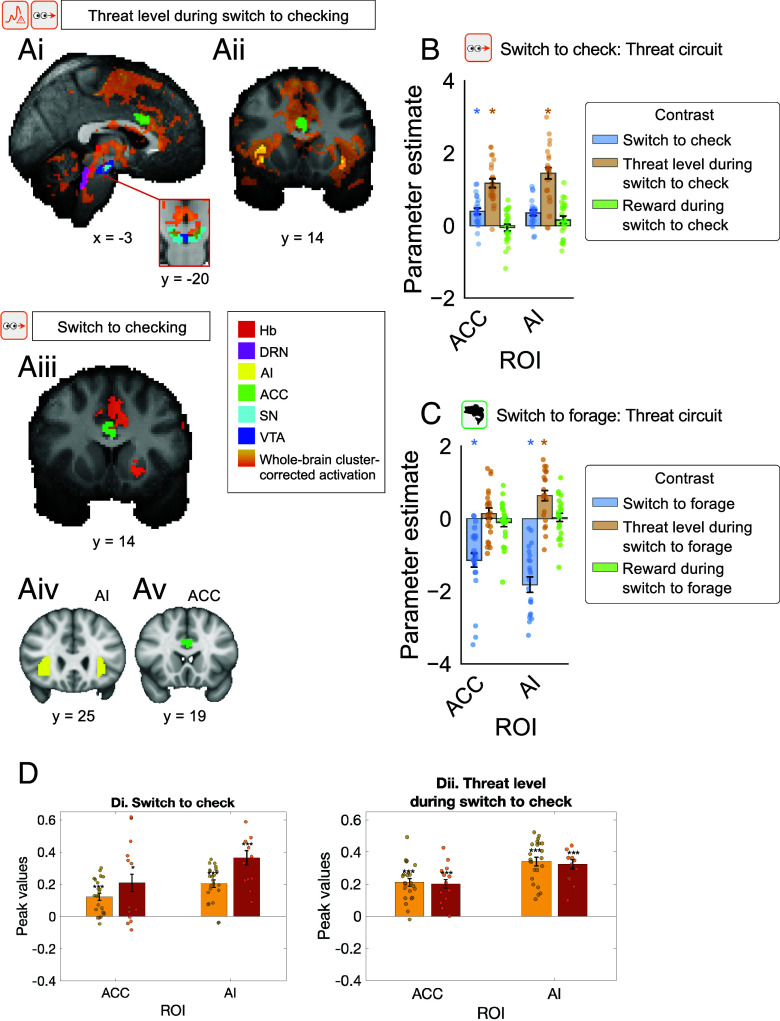
Activity in ACC and AI reflected threat level (time pressure) and switching to checking. A whole brain analysis revealed cortical activity related to threat level (time pressure) when participants were switching to checking and related to the switch to checking per se (whole brain cluster-corrected; Z > 3.1, *P* < 0.001). Threat level (time pressure)-related activity is illustrated on a midline sagittal section (*A*-i) and coronal section (*A*-ii), and activity related to the switch to checking is shown on a coronal section (*A*-iii). The AI and ACC ROIs are illustrated in panels *A*-iv and *A*-v. Activity from these ROIs related to threat level (time pressure), reward rate, and switching is illustrated during switching to checking (*B*) and switching to foraging (*C*). Data in panels *A*–*C* are from the pre-PD phase but the same switching-to-check and threat level activity patterns were apparent in the same ROIs in both pre- (yellow) and post-PD (brown) phases (*D*).

**Fig. 5.**
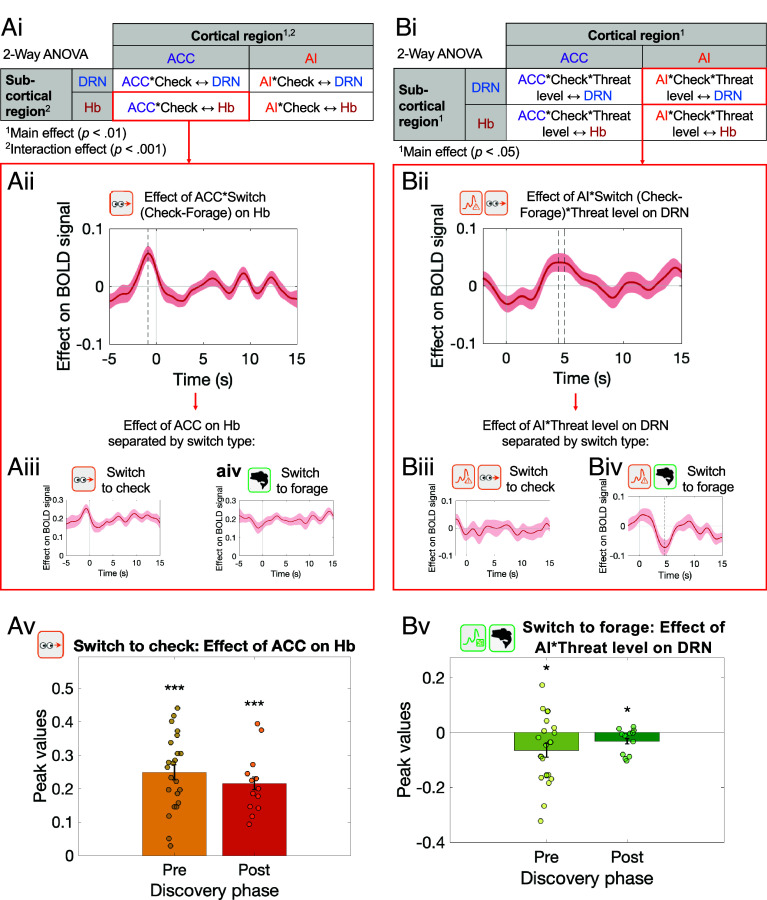
Interactions between cortical areas, Hb, and DRN as a function of threat level and switching to check. Interactions between cortical areas ACC and AI and subcortical areas Hb and DRN were modulated as a function of switching to checking (*A*) and as a function of both switching to checking and time pressure (*B*). (*A-*i) Two-way ANOVA showed a main effect of cortical region (ACC/AI; *P* < 0.01) and an interaction between cortical and subcortical regions (Hb/DRN; *P* < 0.001) on the extent to which check switches vs. forage switches moderated functional connectivity. Average peak values were highest for ACC and Hb activity moderated by checking. Other interactions tested in the ANOVA are plotted in *SI Appendix*, Fig. S10 *A*–*C*. (*A*-ii) Functional connectivity between ACC and Hb was significantly moderated by switching to check (*P* < 0.01). ACC activity interacted with Hb activity differently during check switches (*A*-iii) vs. forage switches (*A-*iv). (*A*-v) The critical interaction between ACC and Hb during switching to check (shown in panel *A*-iii) was similar and significant in both pre-PD (yellow) and post-PD (brown) phases. (*B-*i) Two-way ANOVA showed main effects of both cortical (*P* < 0.05) and subcortical (*P* < 0.01) ROIs on the extent to which check switch and time pressure moderated functional connectivity. Average peak values were highest for interactions with AI as the cortical ROI, and for interactions with DRN as the subcortical ROI. Other interactions tested in the ANOVA are plotted in *SI Appendix*, Fig. S10 *D*–*F*. (*B*-ii) A three-way interaction between AI activity, check switch, and time pressure moderated DRN activity (*P* < 0.05). AI and time pressure affected DRN activity differently during check switches (*B*-iii) vs. forage switches when there was a significant reduction in coupling (*B*-iv). (*B*-v) The critical interaction between AI and DRN—a decrease in coupling as a function of both threat level and switching (shown in panel *B*-iv) was similar and significant in both pre-PD (light green) and post-PD (dark green) phases. In summary, all analyses in *A*-i–iv and *B*-i–iv used pre-PD phase data but the critical interactions were replicated in post-PD data (*A*-v; *B-*v). Significance testing on time course data was performed using a leave-one-out procedure on the group peak signal. The dashed line indicates the average time of peaks across which a two-sided Wilcoxon signed rank test was significant. Absence of dashed line indicates nonsignificance.

**Fig. 6.**
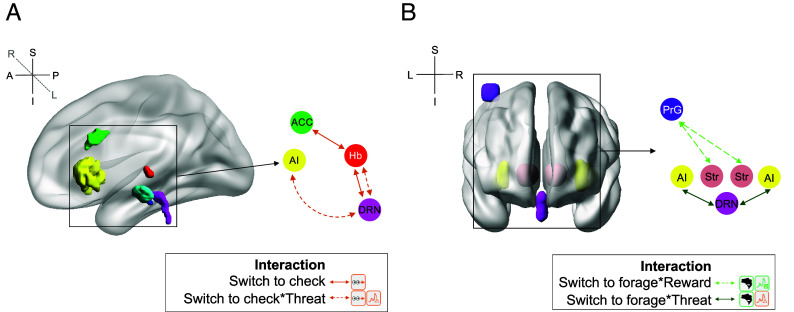
A distributed Neural Network encodes (*A*) threat level (time pressure) and switching to check and (*B*) reward, threat level, and switching to forage. (*A*) In summary, Figs. 2–5 show that in both pre-PD and post-PD phases, activity in ACC, AI, Hb, and DRN reflected threat level during both foraging and checking and also the transition to checking. ACC–Hb and Hb–DRN activity coupling reflected the transition to checking and AI–DRN coupling reflected both the transition away from checking to foraging and threat level. (*B*) In both pre-PD and post-PD phases, activity in precentral gyrus and striatum reflected reward levels and/or the transition to foraging (Fig. 6). Interactions between AI and DRN also reflected the transition away from checking to foraging as a function of threat level (Fig. 5 *B*-iv).

On average, participants’ actions before predator discovery (pre-PD) consisted of approximately equal numbers of checks and forages (44.68 ± 7.80% 55.32 ± 7.80% respectively; Fig. 1*G*). Post-predator discovery (post-PD) participants continued to spend more time on foraging (64.01 ± 6.14%) and checks were now directed toward the known predator direction (10.75 ± 6.95%). The remaining time was spent taking hiding actions (25.24 ± 6.15%; Fig. 1*G*). Our initial analyses, therefore, examined the pre-PD phase when the two key actions, foraging and checking, were both made with a relatively high frequency. However, we subsequently tested the post-PD data and confirmed each of the pre-PD results we report (reviewed in each result figure; Figs. 2–6).

Behavioral analysis showed participants were motivated to avoid threat and able to estimate threat level appropriately. They avoided being eaten by the predator and were only eaten in 1.87 ± 2.19 blocks (6.97% of blocks) on average. In addition, participants adjusted their checking and hiding behavior depending on variation in the threat level as a function of the different predators: When participants knew the fastest predator was present, participants made their first check at an earlier time than when the slowest was present (1.99 s ± 1.15 s vs. 3.75 s ± 2.17 s after block onset, respectively). They also hid at an earlier time (12.25 s ± 1.38 s after the block onset) when the fastest predator was present as opposed to the slowest (19.12 s ± 2.71 s after block onset).

We used a simple regression model to examine the moment-to-moment balance between the two behaviors—checking for threat and foraging—as a function of the two environment features—threat level (time pressure, proximity: *Methods* and *SI Appendix*, Eqs. **S1**–**S3**) and reward rate at the time of action (Fig. 1 *E*, *F*, and *H*). Regression analyses (*SI Appendix*, Eq. **S4** and *Methods*) showed that pre-PD, participants were more likely to check instead of forage as time pressure increased (*t*(22) = 12.94, *P* < 0.0001, *M* = 1.41 ± 0.52) and as reward rate decreased (*t*(22) = −5.38, *P* < 0.0001, *M* = −0.28 ± 0.25; Fig. 1*H*; all *t* tests in *SI Appendix*, Table S1*A*). When we subsequently examined the post-PD behavior, we replicated the same two findings (*SI Appendix*, Table S1*B*); participants were more likely to check instead of forage as threat level increased and as reward rate decreased (*SI Appendix*, Table S1*A*). Post-block stress ratings confirmed that participants experienced greater stress after a fast predator block relative to a slow one (Mann–Whitney U test: Z = 7092.5, *P* < 0.001; Dataset S1). Moreover, we note that in another larger on-line study in which participants provided subjective reports of emotions and arousal levels during the task, again threat level increased anxiety (15).

Next, to gain insights into when critical cognitive processes occurred, we analyzed the time taken between two action types (either foraging or checking; IRTs). In general, IRTs were faster when threat level (time pressure) increased (*t*(22) = 7.30, *P* < 0.001) and when reward level increased (*t*(22) = −4.56, *P* < 0.001; Fig. 1*I* and *SI Appendix*, Table S2). In addition, switching from foraging to checking and vice versa had a significant effect on IRTs (*t*(22) = 10.16, *P* < 0.001). Specifically, it was switching to foraging, rather than simply repeatedly foraging again and again, that was associated with slower IRTs (interaction effect; *t*(22) = −8.07, *P* < 0.001; Fig. 1*I* and *SI Appendix*, Table S2). Mean IRT was slowest when switching from checking to forage and fastest when repeating forages (Fig. 1*J*). Such IRT increases suggest switches between behavioral modes require cognitive resources^21^ (Fig. 1*I* and *SI Appendix*, Table S2). In addition, IRTs between forages became slower as participants approached a switch to checking (*t*(22) = 4.4, *P* < 0.001; Fig. 1*K* and *SI Appendix*, Table S3) suggesting participants prepared to switch to checking prior to actually making the switch. IRT was also significantly slowed by discovery of a new predator (*t*(22) = 6.18, *P* < 0.001; Fig. 1*L* and *SI Appendix*, Table S4). Encountering a new threat requires additional cognitive processing relative to a check that does not reveal new information. In subsequent brain analyses, we focus on understanding these behavioral switches and threat discovery points highlighted by the IRT analysis.

### A Distributed Subcortical Brain Network Monitors Threat and Controls Transitions to Checking for Threat.

The first fMRI analysis focused on activity in the four subcortical ROIs (Hb, SN, VTA, and DRN; Fig. 2*A*) and examined whether it was differentially related to the two key behaviors: switching from foraging to checking for threat (“check switch”) and vice versa (“forage switch”). We examined the two behaviors as a function of the two environmental features that drove these behaviors: time pressure and reward rate. Time-locking to switches in behavior allowed identification of brain activity related to these discrete events despite the free and fast nature of the task (Fig. 1; GLM1; *Methods* and *SI Appendix*, Eq. **S6**, which was structured similarly to the behavioral analyses in *Methods*, *SI Appendix*, Eq. **S4**, Fig. 1*F*). We extracted parameter estimates (β weights from GLM1) in the ROIs shown in Fig. 2*A* (see *Methods* for details about ROIs design and preprocessing).

We then performed a three-way ANOVA on the parameter estimates across the four ROIs. A main effect of switch type revealed that switching to checking was associated with a very different activity pattern than switching to foraging (F(1, 352) = 6.09, *P* = 0.014; blue bars are more positive in Fig. 2 *B* vs. *C*); in general, there was a positive effect on the BOLD signal when switching to checking but a negative one when switching to forage. A main effect of environmental feature showed that threat level effects were stronger than reward rate effects (F(1, 352) = 38.25, *P* < 0.0001; ochre bars are higher than green bars in Fig. 2 *B* and *C*). A two-way interaction between switch type and environmental feature suggested threat level (time pressure) signals were stronger than reward rate signals especially when switching to checking and this pattern was approximately similar across ROIs (F(1,352) = 4.60, *P* = 0.033; ochre bars are especially larger than green bars in Fig. 2 *B* vs. *C*). In summary, the ANOVA revealed that time pressure-related activity, as opposed to reward rate-related activity, was prevalent across all four areas. Time pressure–related activity was always present, even when participants foraged, but it was stronger when participants switched to checking as opposed to foraging (Fig. 2 *B* and *C*). By contrast, in these areas, activity related to reward rate was negligible even at the time of switching to forage (apparent in the near zero reward rate effects in Fig. 2 *B* and *C*). Below (*SI Appendix*, Fig. S2), we discuss reward rate-related activity at the time of switching to foraging found in other structures.

Next, we wanted to test whether threat-related activity in one or other of the subcortical areas correlated with individual differences in checking behavior. To examine this possibility, we tested whether individual variation in the time pressure signal across participants in each subcortical ROI correlated with individual variation in the frequency of checking as indexed by the percentage of responses that were checks. There was indeed a relationship between the strength of the threat level (time pressure) signal and checking frequency across participants in DRN (Pearson’s r = 0.52, *P* = 0.047 after Bonferroni correction for tests across four areas, Fig. 2*D*) but not in the other three areas (all r < 0.42, *P* > 0.176 after Bonferroni correction). We tested whether this DRN-behavior relationship, which we had found in the pre-PD phase of the task, generalized to the post-PD phase; this was the case (Fig. 2*E*; Pearson’s r = 0.41, *P* = 0.025). Again, no significant correlations were found for the three other areas in the post-PD time period (all r < 0.22; all *P* > 0.316). In summary, threat-related activity in DRN was related to switching behavioral focus toward checking for threat stimuli.

Finally, we note that although individual variation in VTA, SN, and Hb activity could not be reliably directly linked to individual variation in the frequency of switching, the main effects of threat level and switching to check that was found in these three nuclei in the pre-PD phase (Fig. 2*B*) were also found in the post-PD task phase (Fig. 2*F*).

### Habenula, DRN, and Their Interaction during Checking and Threat Discovery.

Next, we examined activity at times when checking for threat actually led to predator discovery as opposed to finding no predator. The initial predator discovery event occurs as the end of the pre-PD phase. We focused on Hb and DRN, first, because DRN activity was closely related to switching to checking (Fig. 2 *D* and *E*) and, second, some recent studies have implicated Hb in aspects of information seeking (10, 13) while others have suggested that the Hb is an important source of influence over DRN (7).

Activity in Hb and DRN changed dramatically depending on whether checking failed to identify a predator (Fig. 3 *A* and *B*) or led to predator discovery (Fig. 3 *C*–*F*). A two-way ANOVA applied to the pre-PD data (Fig. 3 *A*–*D*) demonstrated a significant interaction between ROI [Hb, DRN] and predator detection [True, False], F(1,180) = 122.11, *P* < 0.001). Analyses conducted on each area separately revealed Hb activity was significantly higher when checking led to predator detection vs. no detection (compare activity in blue area in Fig. 3*A* with corresponding time in Fig. 3*C*; main effect of predator detection vs. no detection in two-way ANOVA with factor of time period [early vs. late]; F[1,88] = 103.53, *P* < 0.001). DRN activity was significantly lower when checking led to predator detection vs. no detection (compare ochre area in Fig. 3*B* with corresponding area in Fig. 3*D*; main effect of predator detection vs. no detection in two-way ANOVA with additional factor of time period [early vs. late]; F[1,88] = 42.70, *P* < 0.001). Because nondetection checks (Fig. 3*B*), which constitute the bulk of the checks in the pre-PD phase, lack negative DRN modulation, the negative DRN modulation seen when checking led to predator detection (Fig. 3*D*) is not apparent in Fig. 2*B*. While comparison of Fig. 2 *B* and *C*, discussed above, already show a difference between Hb and DRN activity during foraging and checking, consideration of Hb and DRN activity time courses, in the same format as Fig. 3, during forages confirm their difference to the activity patterns seen during checking (*SI Appendix*, Fig. S3).

In the post-PD phase, by definition, the predator is always detected, thus its presence is less surprising; this might lead to a reduction in response in the post-PD phase if the detection response in either Hb or DRN reflects an element of surprise. Nevertheless, a similar pattern to that seen in pre-PD detection trials was seen in post-PD detection trials in both Hb and DRN, albeit with reduced amplitudes (Hb: blue area in Fig. 3*E*; F(1,88) = 52.48, *P* < 0.001; DRN: ochre area in Fig. 3*F*; F(1,88) = 22.36, *P* < 0.001).

Finally, we examined Hb–DRN interactions using psychophysiological interaction (PPI) analyses (22) (Fig. 3 *G* and *H*). Our aim was to examine Hb–DRN interactions as a function of threat level, checking, and predator detection—the three factors identified as influences in Figs. 2 and 3 *A*–*F*. There was a negative 3-way interaction in both pre-PD and post-PD periods (Hb timecourse * Forage/check with threat discovery * threat level, Z = −2.10, *P =* 0.036; in pre-PD phase; Fig. 3*I*; Z = 2.04, *P* = 0.042 in post-PD phase; Fig. 3*J*). This pattern indicates increased negative coupling between Hb and DRN during checking and threat detection as opposed to foraging and it does so as a function of threat level (time pressure). Direction of influence is difficult to ascertain with certainty from BOLD signal analysis but the pattern is consistent with one area inhibiting the other. A negative main effect of Hb activity on DRN activity (*SI Appendix*, Fig. S4 *A* and *B*) is also consistent with inhibition of one area by the other. Hb is known to exert an inhibitory influence over DRN from previous animal studies (7) and the slight timing differences in Hb activation and DRN inhibition (Fig. 3 *C*–*F*) are consistent with Hb inhibition of DRN. Importantly, however, the negative coupling between Hb and DRN as a function of threat level (time pressure) did not occur when checks that did not lead to predator discovery were compared with forages (*SI Appendix*, Fig. S4*E*).

The importance of the information gained by checking is a function of current expectations about threat. However, the amount of information that is gained by checking is also important. While Hb and DRN showed activity related to information-seeking and expectations about threat, they did not reliably encode expectations about the amount of information to be gained by checking. We examined Hb and DRN activity during check switches as a function of the number of directions available for checking, an index of how much information about the environment could be acquired with a single check. Although DRN activity related to pre-PD checks showed a significant relationship with check directions such that lower activity was related to less information being available during checking (*SI Appendix*, Fig. S5*B*), we could not see this relationship in the post-PD phase (*SI Appendix*, Fig. S5*D*), and no such relationship was found in Hb (*SI Appendix*, Fig. S5 *A* and *C*).

Previously (Fig. 2 *D* and *E*) we demonstrated that threat-related activity in DRN was positively correlated with checking frequency. We, therefore, next considered whether variation in the inhibitory interaction between Hb and DRN might, instead, be related to avoiding checking in some way. We found that, indeed, participants in whom there was a larger inhibitory interaction between Hb and DRN took longer to return to checking in the pre-PD phase as a function of threat level (Pearson’s *r* = −0.42, *P* = 0.046).

Unlike in DRN, activity related to switching to check and to threat detection was not seen in VTA (*SI Appendix*, Fig. S6). A pattern of Hb–SN interactions was found when similar tests were performed (*SI Appendix*, Fig. S6) but they occurred later than Hb–DRN interactions. Hb–SN interactions have previously been shown to occur during action inhibition and release (10, 12). In the current context, actions may be inhibited as part of an initial freezing response when the predator is discovered; they continued to be slower during the post-PD phase (Fig. 1*L*). We extended our investigation of the SN further to look at activity related to check switches and threat detection and, in addition, we examined dorsolateral and ventromedial subdivisions of the SN region (dlSN and vmSN, respectively) separately to ensure that we did not overlook any subregion of SN with a clear threat checking and detection signal [previous studies have reported regional variation in SN activity with greater responsiveness to aversive stimuli in more dorsolateral regions (23)]. Checking was associated with an early increase in dlSN and vmSN activity (*SI Appendix*, Fig. S7 *A* and *B*), but after predator detection both subregions showed inhibited activity (*SI Appendix*, Fig. S7 *C* and *D*), particularly as a function of Hb activity, predator detection, and threat level (*SI Appendix*, Fig. S7 *G* and *H*). However, in each case, these patterns were smaller in size (*SI Appendix*, Fig. S7 *C* and *D*) or later (*SI Appendix*, Fig. S7 *G* and *H*) than was the case in DRN (Fig. 3) and again, unlike in DRN, they were not replicated in the post-PD phase (*SI Appendix*, Fig. S7 *E*, *F*, *I,* and *J*). Moreover, although analysis of the dlSN and vmSN regions revealed they generally had a positive relationship with Hb activity (*SI Appendix*, Fig. S8 *A–D*), it did not reveal reliable modulation of subregions activity solely as a function of checking (*SI Appendix*, Fig. S8 *E* and *F*) or as a function of threat level before predator discovery (*SI Appendix*, Fig. S8 *H* and *I*).

The current task was designed to probe threat-related information seeking and detection and is less ideal for assessing how these brain structures respond to surprising rewards. One recent study has demonstrated DRN responses to reward prediction errors (24). A previous study (25), in an analysis focusing on rewarding and aversive events (rather than reward-related and aversive event-related prediction errors) found DRN responses to both rewards and aversive air puffs. While stronger DRN modulation for reward-predicting (as opposed to aversive event-predicting) stimuli was reported, so was greater DRN modulation for the aversive events themselves than to the rewarding events themselves. The current results concur with the suggestion (24) that DRN activity might reflect prediction errors. Just as more surprising rewards (prediction errors) produce a greater response in the VTA, more surprising threat detection produces a stronger response in the DRN; predator detection responses in DRN were stronger at the end of the pre-PD phase than in the post-PD phase and this could be because participants had more information about the predator in the post-PD phase because in this phase they would always have recently observed the predator’s actual proximity to their fish avatar. More broadly, the current findings are consistent with a view of DRN activity that reflects the potential for reward over the longer term (8, 11, 25)—the global reward state—but also the interruption of this state by potentially dangerous or aversive events that require reorienting of behavior. If the global reward state declines then this should drive exploration (24) to identify a better alternative, or if this is not possible, then it might drive a period of quiescence while there is little to be gained from continued reward pursuit (26).

Moreover, previously reported reward-related activity in VTA and threat-related activity in DRN found in the current study are analogous in other ways. For example, activity is more prominent during some behaviors than others; reward coding in VTA is tied to approach behavior as opposed to avoidance behaviour (27, 28). Analogously in the current study, Hb threat-related signals appear when threats are present, but they are most prominent and translated into DRN activity in the context of certain behaviors such as checking for threats as opposed to foraging. The fact that Hb–DRN connectivity reflected threat level is also, in some ways, analogous to the way in which VTA activity reflects reward expectation (29).

In summary, Hb and DRN and their interactions reflect information seeking about threat when participants check, as well as threat expectation (threat level), and threat detection. While the activity patterns have an approximate resemblance to other forms of surprise-related activity previously reported in other brain structures such as VTA, in DRN the activity patterns identified here were linked to information seeking about aversive events. The current experimental design, however, was not optimal for assessing whether activity was related to reward-related information seeking.

### Anterior Cingulate and Anterior Insula Cortex Interact with the Subcortical Areas as a Function of Threat Level and When Switching to Checking.

We assessed whether task-related activity might occur beyond Hb and DRN, in cortical regions such as ACC and AI, with which Hb and DRN interact. We examined activity across the whole brain looking at whole-brain cluster-corrected contrasts (Z > 3.1; *P* < 0.001; Fig. 4*A*; *SI Appendix*, Table S5; and Dataset S2). Despite their small size, the effect of time pressure was robust enough to be seen in Hb, VTA, SN, and DRN, overlapping with the ROIs already reported, even after whole brain cluster-correction. In addition, time pressure-related activity was apparent in ACC and AI (Fig. 4*A*). A second contrast showed that the act of switching to check was also associated with significant activation in ACC (Fig. 2 *B* and *D* and *SI Appendix*, Table S5). For completeness, the effects of time pressure and switching to checking are illustrated for ROIs placed in ACC and AI (Fig. 4 *B* and *C*) from which it can be seen that the two regions hold similar information to one another and to the four subcortical regions previously investigated. Moreover, the same patterns of activity were found when the post-PD data were analyzed (Fig. 4*D*). While we focus here on a limited set of areas—Hb, DRN, ACC, AI—it was also apparent from the whole brain analysis (Fig. 4*A* and *SI Appendix*, Table S5) that threat level (time pressure) during checking and the act of switching to checking was associated with activity in superior colliculus (SC), pulvinar nucleus of the thalamus, and dorsal and ventrolateral parts of the periaqueductal gray (PAG). This is consistent with the suggestion that these brain structures may also mediate fast responses to threat stimuli (30).

Activity in PAG has been closely linked to threat sometimes in studies that used threat stimuli that predicted actual physical pain (31–33). Despite the absence of physical pain delivery in the current study, the time pressure variable was also associated with pre-PD PAG activity (*SI Appendix*, Fig. S9).

### Interactions Across the Distributed Network for Threat Monitoring and Transition to Checking for Threat.

Next, we sought to understand how Hb and DRN activity might emerge within the broader context of cortical activity, in particular, activity in the two cortical areas, ACC and AI, that project to Hb and DRN (17, 18, 34). We examined how these interactions occurred during threat monitoring and at the point of transitioning to check on threats. We therefore again used PPI analyses (22) (*Methods* and *SI Appendix*, Eq. **S8**) to examine task-related interactions between the areas. We found two types of interactions. The first occurred as a function of switching from foraging to checking (Fig. 5*A*). The second also occurred as a function of switching between foraging and checking but, in addition, this interaction reflected threat level (time pressure; Fig. 5*B*).

The first pattern of interaction was evident between ACC and Hb during check for threat as opposed to foraging switches. It was identified by first carrying out a factorial style analysis examining interactions between two cortical regions, ACC and AI, and two subcortical regions, Hb and DRN. A two-way ANOVA revealed a main effect of cortical region (ACC and AI) on the extent to which switching to check for threat moderated functional connectivity with subcortical ROIs Hb and DRN (F(1,180) = 7.03, *P* = 0.009); Fig. 5 *A*-i and *SI Appendix*, Table S6); the post hoc Tukey HSD test found that peak values were greater on average for ACC than AI (*P* adj. = 0.012; 95% CI = [−0.04, −0.01]; and *SI Appendix*, Table S7). There was also a significant interaction between cortical and subcortical regions (two-way ANOVA, F(1,180) = 16.13, *P* < 0.001; Fig. 5 *A*-i; and *SI Appendix*, Table S6), with mean peak values being highest for interaction between ACC and Hb as a function of checking which was significant when tested in isolation (Z = 3.28, *P* = 0.001; Fig. 5 *A*-ii; full PPI analysis results reported in Dataset S3). Stronger ACC activity was associated with stronger Hb activity during check for threat as opposed to forage switches (Fig. 5 *A*-iii and iv illustrate how ACC–Hb interactions differed depending on switch direction). This pattern is specific to ACC–Hb; no evidence was found for similar interactions involving AI and DRN (Fig. 5 and *SI Appendix*, Fig. S10). The significant ACC–Hb switch-to-check effect found in the pre-PD period (Fig. 5 *A*-iii) was also replicated in the post-PD period (Fig. 5 *A*-v).

The second pattern of interaction was like the first in that it reflected interactions between a cortical area and a subcortical area that differed depending on whether participants were switching to checking or switching to foraging. However, this interaction also reflected a third factor, threat level, and it principally emerged between DRN and AI. First, we examined interactions between the two cortical regions, ACC and AI, and the two subcortical regions, Hb and DRN (Fig. 4). There was a main effect of subcortical ROI (Hb and DRN; two-way ANOVA, F(1, 180) = 11.11, *P* = 0.001; *SI Appendix*, Table S8) and a main effect of cortical ROI (ACC and AI; F(1, 180) = 4.22, *P* = 0.04; *SI Appendix*, Table S8) on the extent to which switching to check and time pressure moderated functional connectivity between the four regions. However, the interaction term failed to reach significance, post hoc Tukey HSD tests found that peak values were on average greater for analyses with DRN as the subcortical ROI (*P* adj. = 0.001, 95% CI = [−0.06, −0.01]; *SI Appendix*, Table S9) and greater for analyses with AI as the cortical ROI (*P* adj. = 0.047, 95% CI = [0.0003, 0.05]; *SI Appendix*, Table S10). When we therefore then focused on AI and DRN interactions, there was a three-way interaction between AI activity, threat level (time pressure), and switching to check that modulated DRN activity (Z = 1.98, *P* = 0.048; Fig. 4 *B*-ii, and Dataset S3). This suggests that relatively stronger AI activity was associated with stronger DRN activity as a function of both switch to checking and threat level (Fig. 5 *B*-iii and iv depict how AI activity and time pressure were related to DRN activity differently depending on the direction of switch). Further analysis revealed that the interaction ultimately reflected a negative AI–DRN interaction as a function of threat level when participants switched away from checking to foraging (Fig. 5 *B*-iv). Given that AI–DRN coupling at the time of any switch is, on average, negative (*SI Appendix*, Fig. S11), this pattern means that while strong, negative AI–DRN negative coupling is maintained when the threat level is high and participants are switching to forage, this negative interaction disappears when switching to check. The effect in Fig. 5 *B*-iv is summarized again in Fig. 5 *B*-v; the peak negative interaction identified in each participant (using a leave-one-out analysis) is shown for the pre-PD phase on the left and a significant within-participant replication from the post-PD phase is shown on the right.

In summary, two patterns of cortical–subcortical interaction occurred. The earliest in time reflected positive interactions between ACC and Hb as participants switched to checking. The second interaction occurred between AI and DRN and reflected a negative interaction as a function of time pressure that occurred when participants returned from checking to foraging but which disappeared when participants switched in the opposite direction from foraging to checking. Both patterns are robust; they were identified in both pre-PD and post-PD periods.

### A Distributed Neural Network for the Monitoring of Reward and the Transition to Foraging.

So far, we have focused on proactive switching from foraging to checking as a function of the potential for threat—time pressure—and as a function of reactive detection of threat when the predator was discovered. We next looked for evidence of complementary brain activity mediating behavioral switches in the opposite direction—from checking for threat to foraging. When looking at forage switches, we also considered reward rate because switching from checking to foraging was promoted by higher reward rates (Fig. 1*H*). The network of areas linked to time pressure and check switches exhibited little reward rate-related activity regardless of whether participants were foraging or checking and it was less active during switches to foraging as opposed to checking (Figs. 2 *B*, *C*, and *F* and 4 *B* and *C*).

Reward rate-related activity was found in the whole brain analysis when participants were switching to foraging (GLM1; *SI Appendix*, Fig. S2 and Table S5). It was prominent in a relatively ventral left striatum on either side of the internal capsule and in the cross bridges spanning it (*SI Appendix*, Fig. S2*A*). Additionally, increases in activity related to forage switches and to reward level were found in adjacent parts of the precentral gyrus in dorsal premotor and motor cortices. The reward-related and forage switch–related activity is also summarized in ROIs at each location (*SI Appendix*, Fig. S2 *C* and *D*).

## Discussion

Life in natural environments requires humans and other animals to strike a balance between reward pursuit and threat monitoring. While progress has been made in understanding mechanisms underlying reward- and threat-guided behavior independently, how people and other animals spontaneously switch between the two behavioral modes is not well understood. The present results suggest that behavioral switching between foraging for reward and threat-oriented information seeking depends on a distributed neural circuit that partially overlaps with those concerned with balancing other forms of information seeking and reward-guided behavior (Fig. 6) (1, 13, 21). Monosov (1) and others (35, 36) have argued that a fundamental feature of exploration is whether or not it is instrumental in nature; for example, whether it is directed toward the discovery of rewards or reward-related stimuli. By contrast, noninstrumental exploration reduces uncertainty about the future even when this does not immediately lead to reward. The threat-related information seeking that we found in the current study was not aimed at immediate reward discovery, but it was instrumental in that the information obtained from checking sometimes led to immediate changes in participants’ behavior such as hiding.

In addition to the fundamental importance of such decisions about reward pursuit and threat monitoring for everyday human and animal life, individual variation in how they are taken, as assessed in a large sample, on-line version of the same task, is related to individual variation in anxiety and apathy (15, 37). In the present experimental paradigm, participants freely managed their time and decided themselves when to switch between foraging for rewards vs. checking for approaching predators. Our analysis examined switching points where participants spontaneously changed between foraging and information seeking. We initially focused on the task phase before participants discovered the predator (pre-PD) because during this phase switches to checking and foraging occurred with approximately equal frequency. However, effects found in the pre-PD phase were subsequently replicated within the same participants in the post-PD phase.

Participants tracked the imminence of threat and how recently they had sought information about threats by already checking their environment; increasing time pressure, an estimate of when the predator was likely to appear based on each predator’s speed and the time elapsed since the beginning of the block or the participant’s last check, was associated with switching to check (Fig. 1*H*). As time pressure increased, participants were more likely to check than to forage and as the reward rate increased, they were more likely to forage than to check. It seems unlikely that both the benefits of reward and the potential cost of predation might ultimately be expressed within a single currency. For example, we noticed that, after practicing the task, participants avoided capture by the predator; in general, the participant was caught on only 1.87 ± 2.19 blocks (6.97% of blocks) on average suggesting that they placed a special importance on avoiding predation. However, a future study might employ some version of an approach (12, 38) in which either reward rate or time pressure are manipulated in isolation from one another.

While we found that reward rate and threat level are reliable predictors of whether participants are more likely to forage or to check, it is difficult to make a precise deterministic prediction of exactly when participants will switch from foraging to checking and vice versa. Further investigations of individual differences in task performance in another cohort of participants suggest that the precise timing of switches varies between participants and across similar experimental blocks (15). Some additional variation in behavior can be explained by moment-to-moment changes in emotion and arousal experienced and reported by participants and by individual differences in participants’ psychiatric symptoms and traits. For example, higher self-reported stress is associated with earlier predator discovery times while higher obsessive compulsive traits (39) are associated with altered checking. For example, participants with obsessive compulsive traits check in a disorganized way; they sometimes start rechecking as soon as a check of all directions has been completed (15).

We found evidence of threat (time pressure)-related activity across all the subcortical areas—DRN, SN, VTA, and Hb—that we investigated (Fig. 2). However, DRN was notable; individual variation in DRN’s time pressure activity was robustly linked to individual variation in the frequency of switching to checking (Fig. 2 *D* and *E*). Because of the reciprocal connections between Hb and DRN and the role Hb is thought to have in controlling DRN (7), we examined the time course of both DRN and Hb activity and Hb–DRN interactions during checking when checks led to predator detection or nondetection (Fig. 3 and *SI Appendix*, Figs. S3 and S4). Checking was associated with an increase in DRN activity closely followed by an increase in Hb activity (Fig. 3 *A* and *B*) but when checking led to detection of a predator, initial Hb and DRN activity were followed by a pronounced inhibition in activity in DRN (Fig. 3 *C*-*F*) that was not seen when the predator was not detected. The inhibition was especially marked on the first occasion in a block when the predator was detected at the end of the pre-PD phase (Fig. 3*D*), but it remained present when checks were made in the post-PD phase when the predator would have been observed to be closer with each check that was made (Fig. 3*F*). Variation in the degree of inhibition across participants was correlated with variation across participants in the length of time it took participants to make their first check. Unlike in the pre-PD phase, when Hb activity occurred only when checking, positive Hb activity continued to be present during both checks and forages in the post-PD phase but consistent inhibition of DRN activity only occurred when participants checked and detected the predator. Finally, the checking- and predator detection–related activity in Hb and DRN, and the coupling between them, reflected not just the checking action and detection event but also the prior estimate of threat (Fig. 3 *G* and *H*). That Hb might exert an inhibitory influence over DRN is consistent with previous analyses of the regions’ interconnections (7) and also with the finding that some aspects of activity in the two areas manifest in opposite directions under similar conditions (13, 24). However, the finding that Hb activation is only translated into strong DRN inhibition on some occasions, during predator detection, suggests that initial DRN activity levels and other aspects of Hb–DRN connectivity, possibly reciprocal connections from DRN to Hb also determine the final state of the DRN.

Although our focus was on DRN, supplementary analyses of the VTA failed to find evidence of the same pattern of inhibition linked to threat detection; by analyzing each individual participant’s VTA activity time course we were able to identify time points when it was most negative in each person. However, there were points of activity inhibition in VTA they occurred randomly over 6 s in the analysis period across all participants so that no reliable point of inhibition was visible in the group data. (*SI Appendix*, Fig. S6*B*). SN activity more closely resembled DRN activity but the peak time of inhibition was later than in DRN (*SI Appendix*, Fig. S6*A*). A close relationship between VTA activity and reward and SN responsiveness to both rewarding and aversive stimuli has previously been reported in monkeys (23). The present results are consistent with the notion that SN may respond to both positive and negative events, even if there are clearer differences between VTA and DRN.

In the cortex, ACC and AI also carried threat (time pressure)-related activity and ACC and AI activity increased on checking and decreased when participants returned to foraging. While additional routes of interaction may exist between these cortical regions and Hb and DRN, it is known, albeit sometimes only from rodent studies, that the cortical regions receive direct projections from DRN and in turn project to Hb and DRN (17, 18, 34, 40). Robust patterns of interaction were also found between Hb and ACC depending on whether participants were switching to foraging or checking; there was pronounced positive interaction between ACC and Hb during checking (Fig. 5 *A*-ii, iii, and v). AI–DRN interactions were especially specific and occurred as a function of both time pressure and switching to check (Fig. 5 B-ii, iv, and v). These interactions reflected a period of negative coupling when participants switched back to checking to foraging which was absent when switching in the opposite direction—from checking to foraging.

In some cases, reward seeking and threat checking may not be mutually exclusive behaviors. For example, rodents continuously monitor the upper field from where predatory threats are common and this may continue even during foraging. Predator avoidance responses to looming (expanding) visual stimuli in the upper visual field are mediated by the SC (41). A possibly similar sensitivity to looming stimuli in the upper visual field is present in humans (42). However, once such threats are detected there is, as in the current task, a need to disengage from reward-oriented behavior and to respond to the threat. For such looming upper visual field stimuli, this appears to be linked to a pathway from the SC to inhibitory GABA-ergic neurons in the VTA (43). Moreover, some of the same cortical regions that were identified with switching between reward-oriented foraging and threat-oriented checking in the current study may also inhibit collicular responses to threat stimuli; the ACC neurons that respond to danger signals send excitatory projections to the SC while ACC neurons that respond to safety signals project to the zona incerta which, in turn, sends an inhibitory projection to the SC (44).

It has been suggested that DRN encodes a prediction error signal in response to aversive stimuli that might complement one for tracking rewards in dopaminergic nuclei (45). More recent research, however, has suggested that while DRN activity may indeed reflect aversive events it also reflects appetitive ones and that a prolonged increase in serotonin increases learning signals for aversive and appetitive stimuli (8, 11, 24, 25, 46). Nevertheless, the induction of activity in DRN does not appear to be rewarding in a simple way; but instead, it leads to changes in the choices animals make as a function of both costs and benefits; for example, after DRN stimulation, mice become more likely to wait through a delay to obtain a reward (47). The current results suggest that DRN’s mediation of patience and motor inhibition when deciding how long to wait for a reward may be part of a more general role DRN has in balancing the impact that aversive and appetitive prospects have on behavior. One possibility, therefore, is that DRN has a general role in tracking both rewarding and aversive features of the environment, with the latter especially salient in the current study, in order to redirect the motivational focus for behavior toward or away from reward. In the present study, redirection occurs between reward- and threat-related behavioral orientations. In another, recent study from our laboratory we have observed DRN activity as macaques switch between reward-related motivation and inaction (26). Relatedly, activity patterns in zebrafish DRN can be interpreted as being related to switches between reward-guided motivation and exploration (48). The current results suggest these insights from fish, rodents, and nonhuman primate studies may be useful in understanding how humans decide when and how frequently to direct behavior to check and seek more information about the environment as opposed to simply focusing on reward pursuit.

More broadly, the current findings are consistent with a view of DRN activity that reflects the potential for reward over the longer term (8, 11, 25)—the global reward state—but also the interruption of this state by potentially dangerous or aversive events that require reorienting of behavior. If the global reward state declines then this should drive exploration (24) to identify a better alternative, or if this is not possible, then it might drive a period of quiescence while there is little to be gained from continued reward pursuit (26).

## Methods

### Subjects.

24 healthy adult participants (15 females), aged 18 to 35, completed the study. Participants were paid £10 and £15 per hour for the online and scan sessions respectively, plus a performance-dependent bonus (£5.10 ± 0.86). Ethical approval was given by the Oxford University Central University Research Ethics Committee (Ref-Number MSD-IDREC-R55856/RE006). All participants provided informed consent before the experiment. One participant was excluded from all analyses because they did not make enough check actions to compute all regressors of interest in the model. Behavioral data from all other participants were included in analyses.

### Task.

We designed a gamified foraging task in which participants freely made a series of choices with the goal of earning as much money as possible. During the task (Fig. 1 *A*–*D*) participants used arrow keys to control an animated fish in an ocean environment where there was rewarding food (later translated to a bonus payment), threatening predators (leading to large point loss if they “caught” the fish), and a hiding space (where participants could hide from the predators). Participants chose freely between three actions: “forage” for food, check for predators, and hide in a safe space. Participants completed an online version of the task, including instructions, three sample blocks, and a quiz to test their knowledge, before arriving for the scan. Each participant received one of two task schedules, each having the same scheduled blocks but in a different, randomized order. See *SI Appendix*, *Supplementary Methods* for further details about the task structure and behavioral analysis.

### Neural Recording.

We used ultra-high field functional MRI (7T fMRI) to identify brain activity corresponding to task behavior.

#### Data acquisition.

We used a Siemens 7T MRI scanner to collect structural and functional MRI. High-resolution functional data were acquired using a multiband gradient-echo T2* echo planar imaging (EPI) sequence with a 1.5 × 1.5 × 1.5 mm resolution; multiband acceleration factor 3; repetition time (TR) 1,962 ms; echo time (TE) 20 ms; flip angle 68°; and a GRAPPA acceleration factor 2. Field of view (FOV) covered the whole brain with axial orientation and a fixed angulation of −30° (anterior-to-posterior phase encoding direction; 96 slices). In addition, a single-measurement, whole-brain, functional image with similar orientation (expanded functional image) was acquired prior to the main functional image and later used to improve registration of the main functional image. Structural data were acquired with a T1-weighted MP-RAGE sequence with a 0.7 × 0.7 × 0.7 mm resolution; GRAPPA acceleration factor 2; TR 2,200 ms; TE 3.02 ms; and inversion time (TI) 1,050 ms. To correct for magnetic field inhomogeneities a field map was acquired with a 2 × 2 × 2 mm resolution; TR 620 ms; TE1 4.08 ms; TE2 5.10 ms. Finally, cardiac and respiratory measurements were collected using pulse oximetry and respiratory bellows to regress out the effect of physiological noise in the functional data (49–51). Functional images were normalized, spatially smoothed (Gaussian kernel with 3 mm full-width half-maximum), and temporally high-pass filtered (cut-off of 100 s). Motion correction was performed using MCFLIRT (52) and separation of the brain from nonbrain matter was performed using the brain extraction tool (BET) (53). Registration of functional images into Montreal Neurological Institute (MNI) space was carried out in three stages: First, the main functional image was registered to the expanded functional image using FMRIB’s linear image registration tool (52, 54) with three degrees of freedom (translation only); second, the main functional image was registered to the individual structural image using boundary-based registration (BBR) (55), incorporating Fieldmap correction; and third, the individual structural image was registered to standard space by using FMRIB’s nonlinear image registration tool (FNIRT) (56). See *Whole-brain analyses, ROI time course analyses,* and *Post-PD phase analyses* in *SI Appendix*, *Supplementary Methods* for details of neural recording analysis.

## Supplementary Material

Appendix 01 (PDF)

Dataset S01 (CSV)

Dataset S02 (CSV)

Dataset S03 (CSV)

## Data Availability

Anonymized behavioral and fMRI data have been made available (57). We are including fMRI data extracted from all the brain regions presented in the manuscript and are doing so for each participant, but we do not include any MRI data from which it might be possible to identify individual participant faces as is the standard in the field.
