# Supplementary material for: A distributed subcortical circuit linked to instrumental information-seeking about threat
Source: Proc Natl Acad Sci U S A. 2025 Jan 15;122(3):e2410955121. doi: 10.1073/pnas.2410955121 (PMC11761969; doi:10.1073/pnas.2410955121)
Supplement: Supplementary file 1 — Appendix 01 (PDF) [file pnas.2410955121.sapp.pdf]

Supplementary Figures

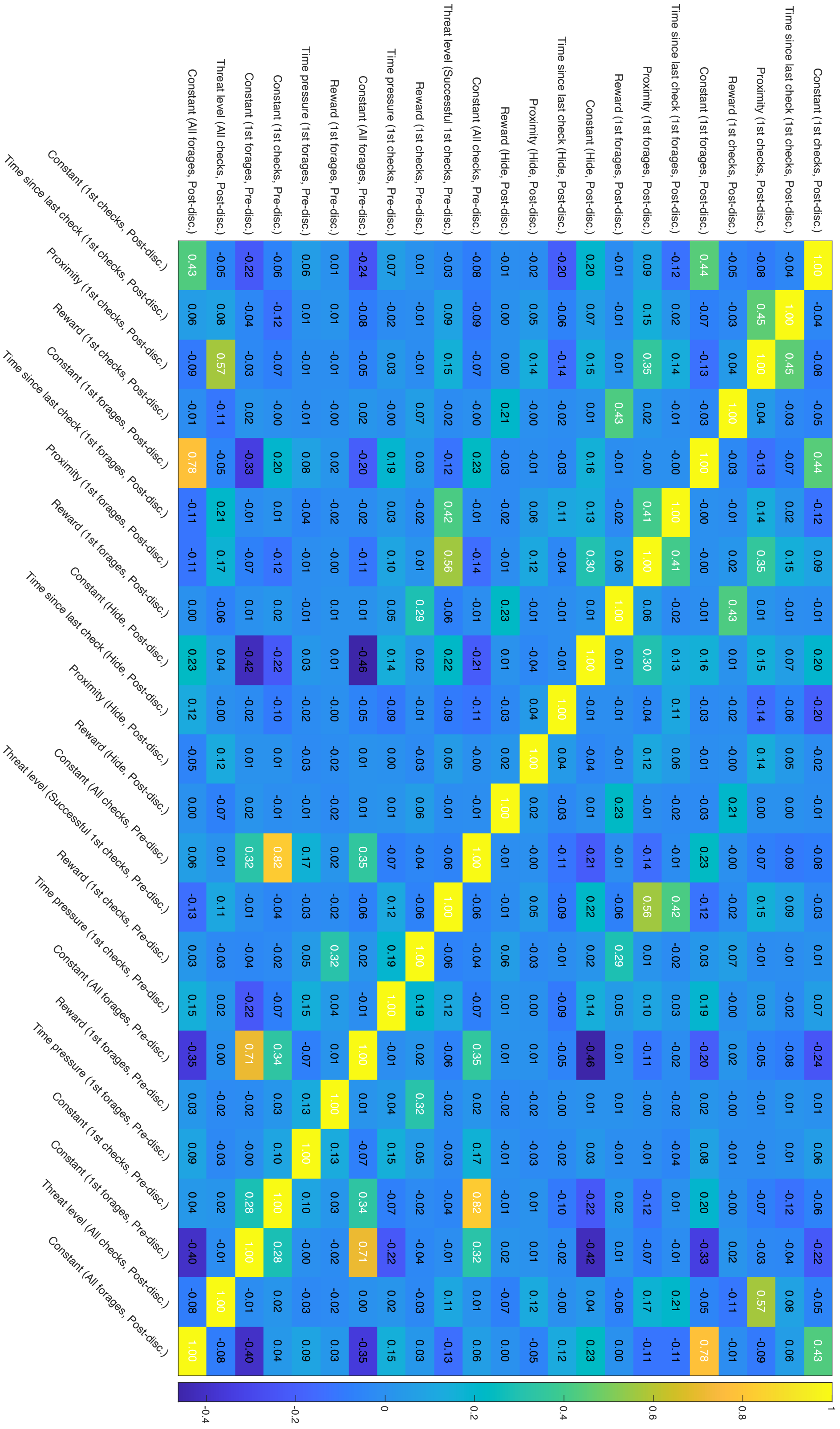

Figure S1. Correlation matrix for regressors included in the fMRI analysis, averaged across all participants. *Constant terms from the regression model denoting the first check in a series of checks and the subsequent checks in the same series were correlated as were the first forage and subsequent forages in a series of forages. However, there were no other correlations affecting the interpretation of key analyses.*

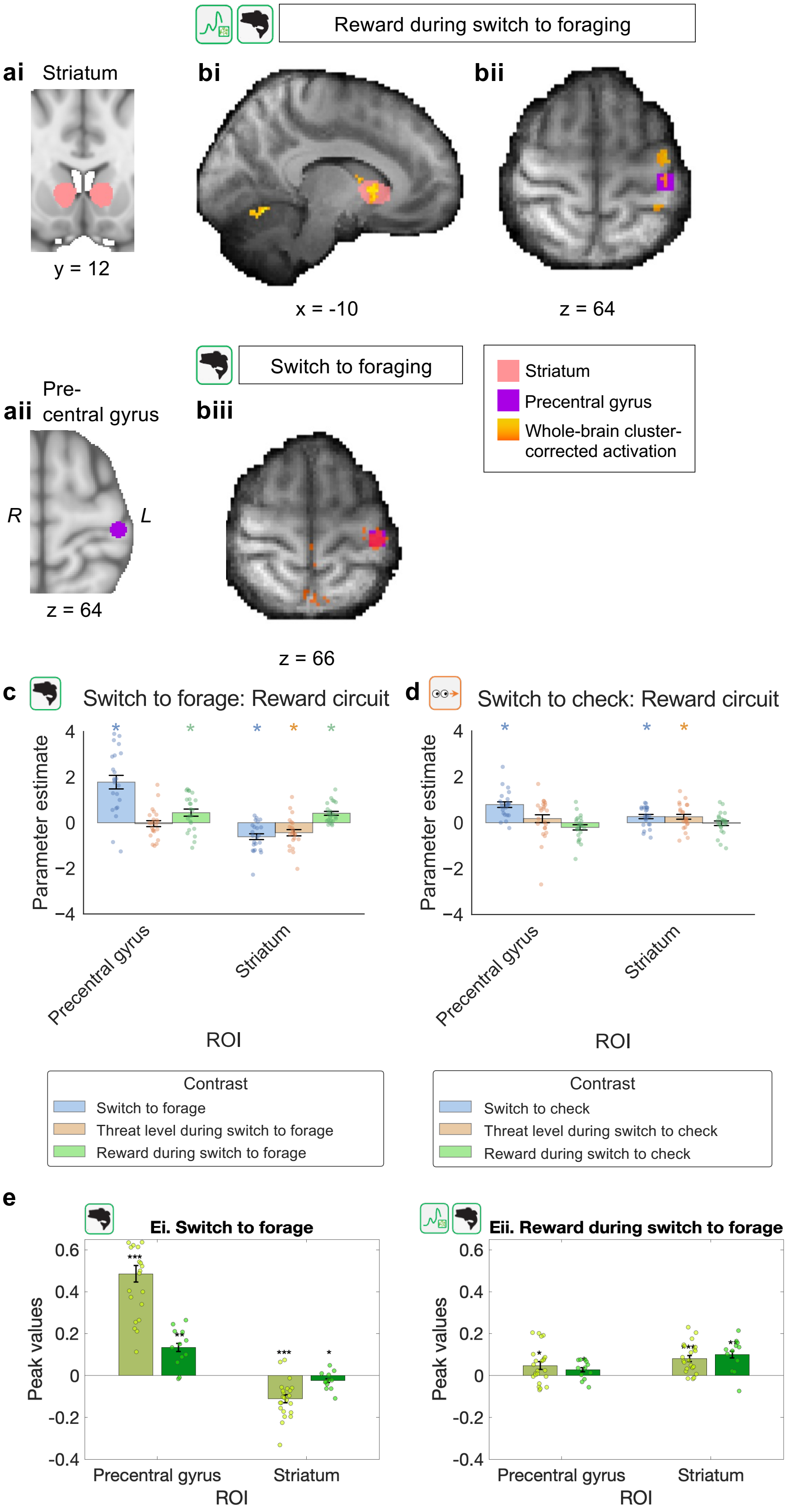

Reward during switch to foraging
ai Striatum
y = 12
bi
x = -10
bii
z = 64
Switch to foraging
aii Pre-central gyrus
R
L
z = 64
biii
z = 66
Striatum
Precentral gyrus
Whole-brain cluster-corrected activation
c Switch to forage: Reward circuit
Parameter estimate
Precentral gyrus
Striatum
ROI
d Switch to check: Reward circuit
Contrast
Switch to forage
Threat level during switch to forage
Reward during switch to forage
Contrast
Switch to check
Threat level during switch to check
Reward during switch to check
e
Ei. Switch to forage
Eii. Reward during switch to forage
Peak values
Precentral gyrus
Striatum
ROI

Figure S2. Activity related to switching to foraging, time pressure, and reward. *A-B) Whole-brain analysis (whole-brain cluster-based correction, Z>3.1, p<0.001) revealed activity in the striatum (Ai, Bi) and precentral gyrus (Aii, Bii) was correlated with reward during switching to forage (B). In addition, activity in the precentral gyrus was positively correlated with the act of switching to forage itself (Biii). Legend indicates color-coding of whole-brain cluster corrected activations and ROI masks. C-D) Parameter estimates (mean and standard error) associated with switches to foraging and checking as well as environmental variables (time pressure, reward) during each switch. There was a positive reward signal in the precentral gyrus and striatum during switches to foraging (C) that was greater than the equivalent in switches to checking (D). Significance testing on time course data was performed using a leave-one-out procedure on the group peak signal. Dashed line indicates the average time of peaks across which two-sided Wilcoxon signed rank test was significant. *= p < 0.001 after whole-brain cluster correction.*

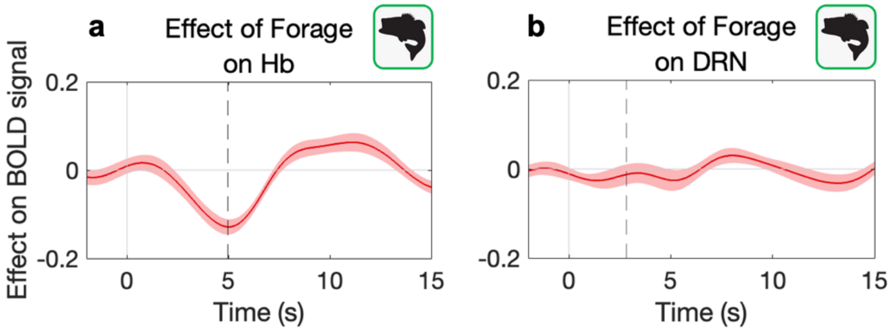

Figure S3. *The increases in Hb and DRN seen during checking even in the absence of predator detection (3A, B) in the pre-PD phase were absent from Hb (A) and DRN (B) during foraging.*

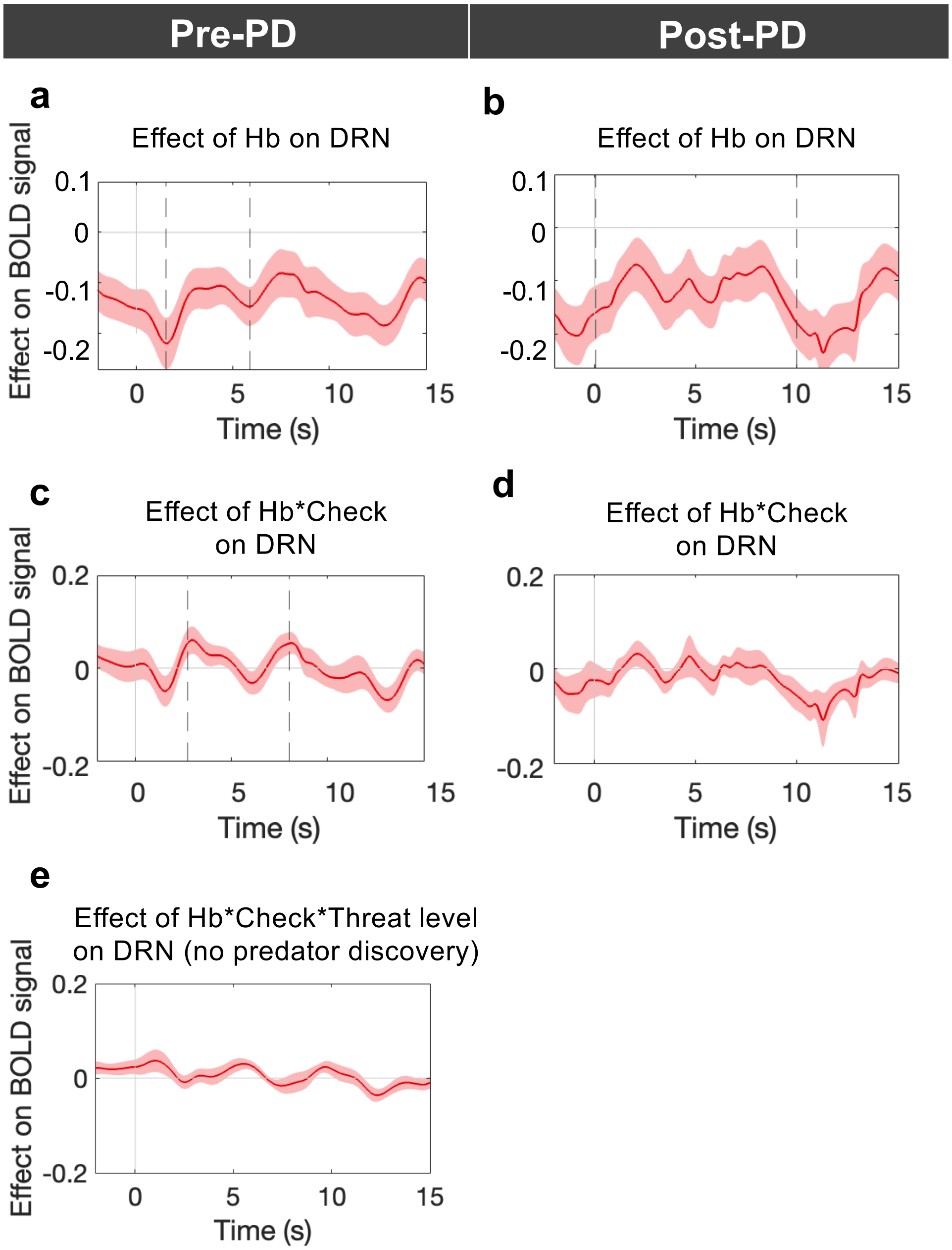

Figure S4. Activity in Hb and DRN during checking. *To guide interpretation of the PPI results shown in figure 3G-H, we looked at the general impact of Hb activity on DRN (the main effect of Hb activity on DRN in the PPI analysis). In both pre-PD (A) and post-PD (B), Hb activity was associated with a sustained reduction in DRN activity. However, when we focussed specifically on checking trials, it was apparent that a more complex series of facilitatory and inhibitory periods of interactions occurred between Hb and DRN in both pre-PD (C) and post-PD (D) periods. (E) A PPI analysis examining the interaction between Hb activity, threat (time pressure), on checking trials that led to no predator discovery versus forage trials revealed no period of significant negative interaction between Hb and DRN. By contrast, when checking led to predator detection, a similar analysis identified a marked period of inhibition (Fig.3G,H).*

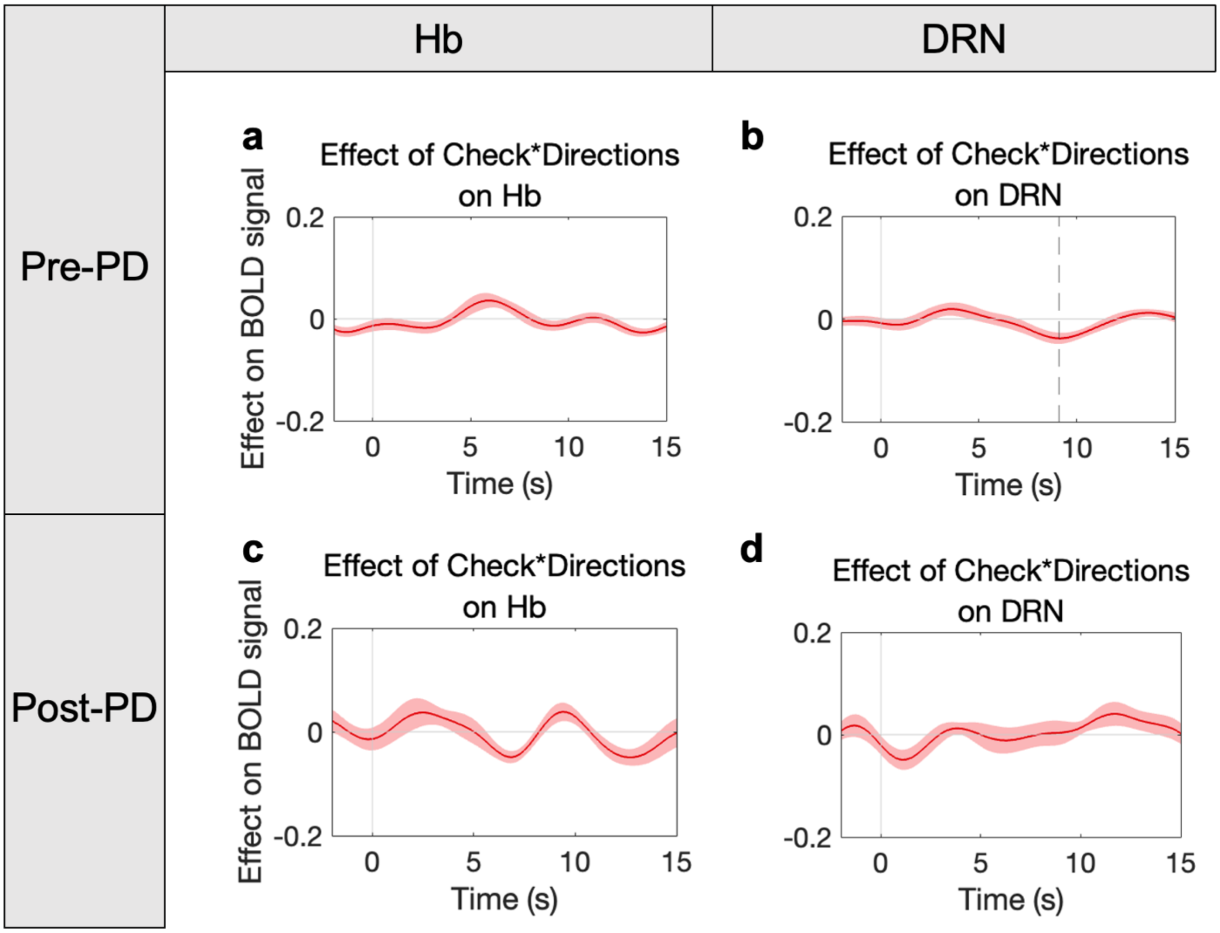

Figure S5. *Activity in Hb and DRN during switch to check as a function of the amount of information obtained by checking. We examined Hb and DRN activity during switches to check as a function of the number of directions available for checking. When more directions were available for checking (up to 4), each check revealed a smaller view of the environment and therefore less information was acquired. DRN activity related to pre-PD checks showed a significant relationship with check directions such that lower activity was related to less information being available during checking. However, we could not see this relationship in the post-PD phase, and no such relationship was found in Hb.*

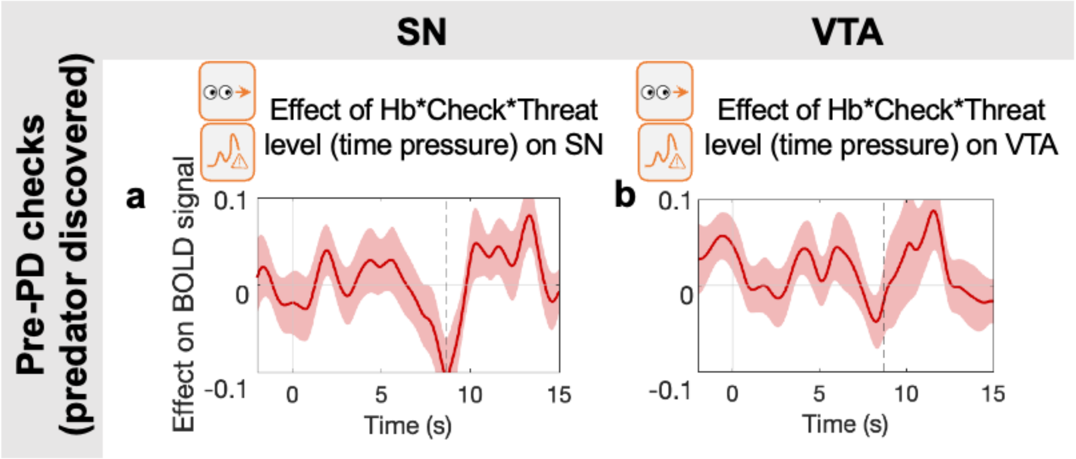

Figure S6. *Timing of inhibition in SN and VTA. PPI analyses identified a period of inhibitory interaction between Hb and DRN when checking led to threat discovery (Fig.3C-F). (A) This period of inhibitory interaction was identified at a consistent time across participants in DRN. A similarly consistent period of inhibitory interaction was identified between Hb and SN but it occurred several seconds later (B) No consistent period of inhibitory interaction was identified between Hb and VTA and any periods identified in individual participants occurred later than between Hb and DRN. Hb-SN interactions have previously been reported during action inhibition and release (1, 2). In the current task, actions may be inhibited as part of a freezing response when the predator is discovered at the end of the pre-PD phase and while it remains present in the post-PD phase (Fig. 1K). Small SN activity decreases were also found during forages in the post-PD phase (Supplementary Fig.S3).*

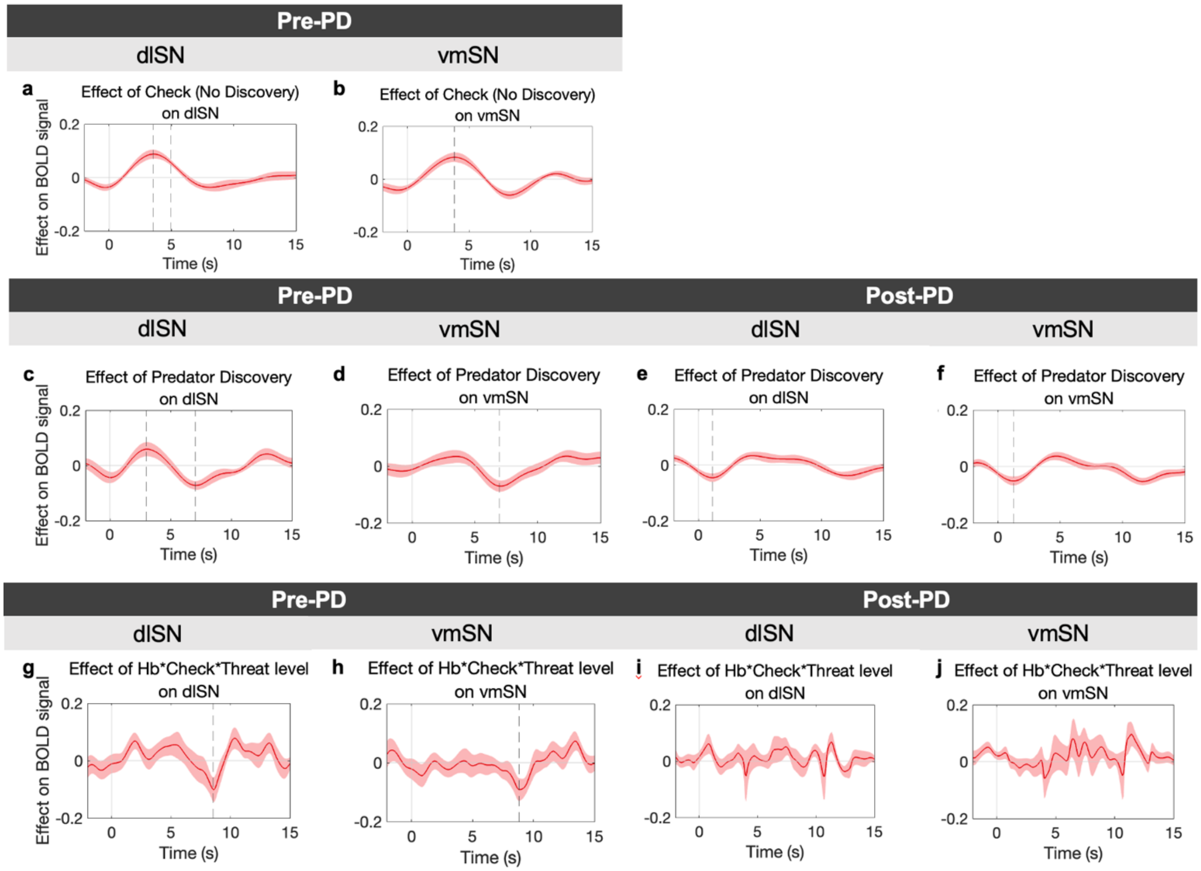

Figure S7. *Dorsolateral and ventromedial SN subregions (dlSN and vmSN) showed activity related to switching to check and threat detection. Checking was associated with an early increase in dlSN and vmSN activity (A, B), but after predator detection both subregions showed inhibited activity (C, D), particularly as a function of Hb activity, predator detection, and threat level (G, H). However, in each case, these patterns were smaller in size (C, D) or later (G, H) than was the case in DRN (figure 3) and again, unlike in DRN, they were not replicated in the post-PD phase (E, F, I, J).*

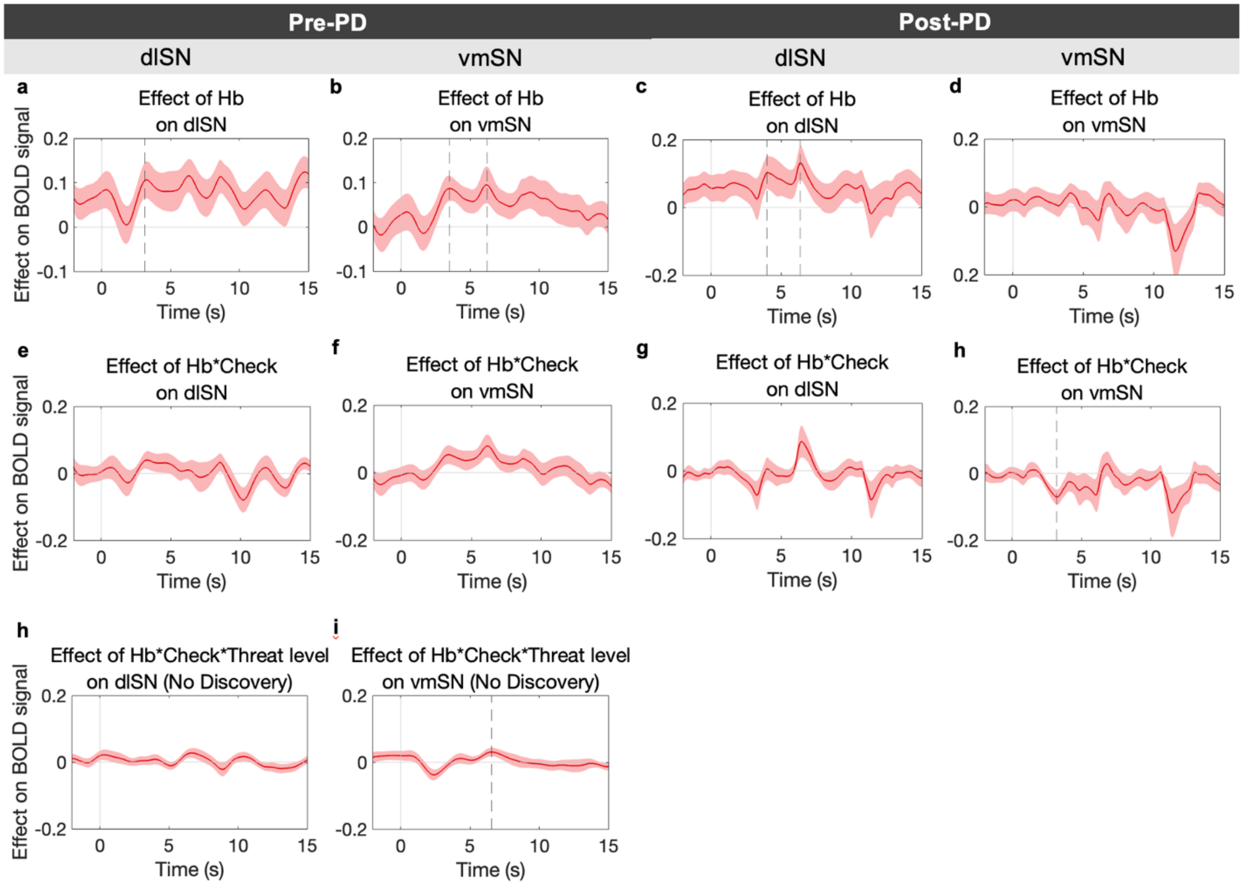

Figure S8. *Relationships between activity in Hb and SN subregions, dlSN and vmSN. Hb activity generally had a positive relationship with dlSN and vmSN activity (A, B, C), although this was more difficult to detect in vmSN during the post-PD phase (D). However, unlike DRN, Hb did not modulate the activity of the SN subregions as a function of switching to check (E, F, G, H) or as a function of threat level when a predator was not discovered (H, I).*

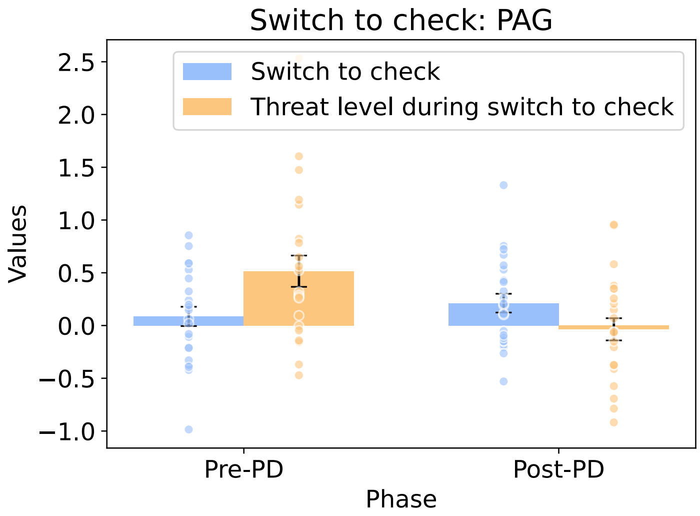

Figure S9. *Activity in periaqueductal grey (PAG). Activity in PAG has been closely linked to threat sometimes in studies that used threat stimuli that predicted actual physical pain (20–22). Despite the absence of physical pain delivery in the current study, the time pressure variable was also associated with PAG activity. We did not find an association between threat level and PAG in the post-PD phase.*

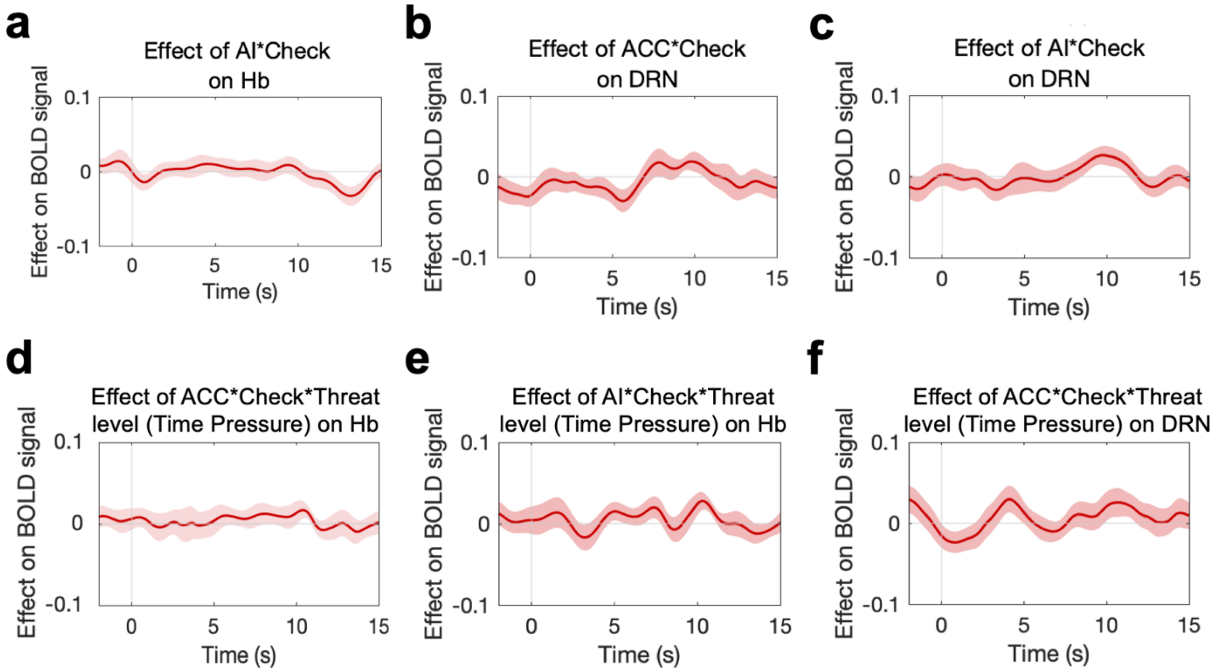

Figure S10. *Interactions between activity in cortical areas ACC and AI and subcortical areas Hb and DRN as a function of transitioning to checking (A-C) and as a function of both transitioning to checking and threat level (as indexed by time pressure) (D-F). Peak values in A-C were entered into the two-way ANOVA summarized in Fig. 5Ai and reported in Table S6. Peak values in D-F were entered into the two-way ANOVA summarized in Fig. 5Bi and reported in Table S6. In general, all the interaction patterns shown here, in contrast to those shown in Fig. 5Aii and Fig. 5Bii are non-significant.*

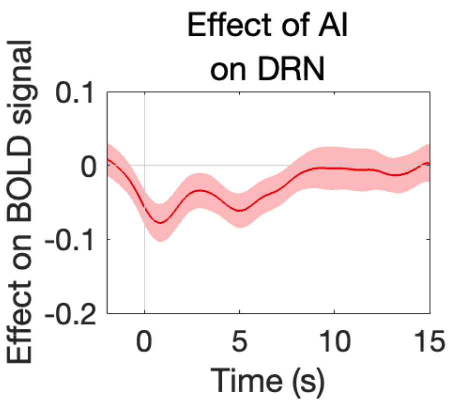

Figure S11. *Interactions between AI and DRN were, in general, negative at the point of switching.*

Supplementary Tables

Table S1.

Two-Tailed Single Sample T-Tests Across Regression Coefficients Showing Effects of Contextual Factors on Action Selection (Related to Fig. 1H)

			95% CI		Degrees of		
Parameter	*P* value	T statistic	Lower	Upper	freedom	Mean	SD
**Table S1.a**							
[1]Model: $Check\ (Pre\ disc.phase) \sim 1 + \beta_1 * Reward + \beta_2 * TimePressure$							
[1]Reward	0	-5.38	-0.39	-0.17	22	-0.28	0.25
[1]Time pressure	0	12.94	1.18	1.64	22	1.41	0.52
**Table S1.b**							
[2]Model: $Check\ (Post\ disc.phase) \sim 1 + \beta_1 * Reward + \beta_2 * TimeSinceLastCheck + \beta_3 * Proximity$							
[2]Reward	0.242	-1.2	-0.19	0.05	22	-0.07	0.27
[2]Time since last check	0	5.24	3.24	7.49	22	5.37	4.91
[2]Proximity	0	-7.72	-0.15	-0.09	22	-0.12	0.08
**Table S1.c**							
[3]Model: $Forage\ (Post\ disc.phase) \sim 1 + \beta_1 * Reward + \beta_2 * TimeSinceLastCheck + \beta_3 * Proximity$							
[3]Reward	0.256	1.17	-0.03	0.09	22	0.03	0.14
[3]Time since last check	0	-8.72	-7.02	-4.32	22	-5.67	3.12
[3]Proximity	0	-14.61	-0.43	-0.32	22	-0.37	0.12
**Table S1.d**							
[4]Model: $Hide\ (Post\ disc.phase) \sim 1 + \beta_1 * Reward + \beta_2 * TimeSinceLastCheck + \beta_3 * Proximity$							
[4]Reward	0.972	0.04	-0.07	0.07	22	0	0.16
[4]Time since last check	0.018	2.56	0.37	3.56	22	1.97	3.68
[4]Proximity	0	16.77	0.67	0.86	22	0.76	0.22

Table S2.

Two-Tailed Single Sample T-Tests Across Regression Coefficients Showing Effects of Transition, Action Type, and Contextual Factors on Inter-Response Times (IRTs) (Related to Fig. 1I)

Parameter	*P* value	T statistic	95% CI Lower	95% CI Upper	Degrees of freedom	Mean	SD
Transition	0	10.16	0.18	0.27	22	0.22	0.1
Check	0	4.23	0.14	0.41	22	0.27	0.31
Transition*Check	0	-8.07	-0.31	-0.18	22	-0.24	0.14
Time pressure	0	7.3	0.13	0.23	22	0.18	0.12
Reward	0	-4.56	-0.06	-0.02	22	-0.04	0.04
Time elapsed	0	-6.94	-0.06	-0.03	22	-0.05	0.03
Block	0.043	-2.15	-0.02	0	22	-0.01	0.02

Note. Model: $RT\ (Pre\ disc.phase) \sim 1 + \beta_1 * transition + \beta_2 * check + \beta_3 * (transition * check) + \beta_4 * timePressure + \beta_5 * reward + \beta_6 * timeElapsed + \beta_7 * block$

Table S3.

Two-Tailed Single Sample T-Tests Across Regression Coefficients Showing Effects of Approaching a Switch to Checking on Inter-Response Times (IRTs) between Forages (Related to Fig. 1K)

Parameter	*P* value	T statistic	95% CI Lower	95% CI Upper	Degrees of freedom	Mean	SD
Time pressure	0	7.24	0.2	0.37	22	0.28	0.19
Reward	0	-5.94	-0.12	-0.06	22	-0.09	0.07
Time elapsed	0	-5.22	-0.1	-0.04	22	-0.07	0.06
Proximity to check switch	0	4.4	0.06	0.15	22	0.1	0.11
Block	0.156	-1.47	-0.02	0	22	-0.01	0.03

Note. Model: $RT\ (Pre\ disc.phase) \sim 1 + \beta_1 \text{timePressure} + \beta_2 reward + \beta_3 timeElapsed + \beta_4 proxToCheckSwitch + \beta_5 * block$

Table S4.

Two-Tailed Single Sample T-Tests Across Regression Coefficients Showing Effects of Predator Discovery on Inter-Response Times (IRTs) (Related to Fig. 1L)

			95% CI				
Parameter	*P* value	T statistic	Lower	Upper	Degrees of freedom	Mean	SD
First action post-predator discovery	0	6.18	0.07	0.13	22	0.1	0.08
Reward	0.011	-2.79	-0.06	-0.01	22	-0.04	0.06
Block	0.03	-2.32	-0.02	0	22	-0.01	0.02
Time elapsed	0	-5.92	-0.05	-0.03	22	-0.04	0.03
Pre-discovery phase (vs. post)	0.004	-3.17	-0.08	-0.02	22	-0.05	0.08

Note. Model: $RT\ (Pre + post\ disc.phases) \sim 1 +\ \beta_1 * postPredatorDisc +\ \beta_2 * reward + \beta_3 * block + \beta_4 * timeElapsed + \beta_5 * preDiscPhase$

Table S5.

Whole-Brain Cluster-Corrected Activations and their Peak Values within ROIs

Time-locked action	Parameter	Cluster type	ROI	Peak MNI coord.	Peak Z	No. of voxels*	Cluster index
Check switch	Constant	Act.	Pulvinar	(-14, -22, 12)	4.44	38	1
Check switch	Constant	Act.	Striatum	(-16, 14, -2)	3.67	4	3
Check switch	Constant	Act.	ACC	(-2, 12, 32)	3.94	21	7
Check switch	Constant	Act.	Precentral gyrus	(-40, -24, 64)	4.54	23	7
Check switch	Threat level	Act.	ACC	(0, 16, 28)	5.30	95	2
Check switch	Threat level	Act.	DRN	(2, -30, -16)	4.43	22	2
Check switch	Threat level	Act.	Hb	(-4, -24, 0)	4.35	26	2
Check switch	Threat level	Act.	AI	(30, 22, -10)	6.00	422	2
Check switch	Threat level	Act.	PAG	(6, -30, -10)	4.28	37	2
Check switch	Threat level	Act.	Pulvinar	(8, -24, 14)	4.81	88	2
Check switch	Threat level	Act.	SC	(4, -28, -4)	4.61	50	2
Check switch	Threat level	Act.	SN	(-6, -20, -12)	4.19	57	2
Check switch	Threat level	Act.	Striatum	(-14, 18, 4)	4.18	24	2
Check switch	Threat level	Act.	VTA	(-4, -20, -14)	3.86	5	2
Forage switch	Constant	Act.	Precentral gyrus	(-40, -30, 64)	6.19	44	4
Forage switch	Constant	Deact.	Striatum	(8, 12, 2)	6.17	168	9
Forage switch	Constant	Deact.	DRN	(0, -32, -20)	4.46	34	12
Forage switch	Constant	Deact.	Hb	(-4, -24, 0)	4.97	31	12
Forage switch	Constant	Deact.	PAG	(4, -30, -6)	4.65	48	12
Forage switch	Constant	Deact.	Pulvinar	(10, -24, 0)	4.69	15	12
Forage switch	Constant	Deact.	SC	(-4, -30, -4)	5.51	77	12
Forage switch	Constant	Deact.	SN	(8, -26, -16)	4.26	58	12
Forage switch	Constant	Deact.	VTA	(-4, -12, -10)	3.76	6	12
Forage switch	Constant	Deact.	AI	(-30, 20, 10)	6.11	419	13
Forage switch	Constant	Deact.	ACC	(-2, 16, 24)	5.41	97	14
Forage switch	Threat level	Act.	Hb	(6, -24, 0)	3.74	6	1
Forage switch	Threat level	Act.	PAG	(4, -30, -10)	4.24	18	1
Forage switch	Threat level	Act.	Pulvinar	(10, -24, 0)	3.92	3	1

Forage switch	Threat level	Act.	SC	(4, -30, -4)	3.92	13	1
Forage switch	Threat level	Act.	AI	(32, 28, 4)	4.78	144	8
Forage switch	Threat level	Deact.	Striatum	(10, 12, -4)	4.54	57	6
Forage switch	Reward	Act.	Striatum	(-12, 14, 4)	4.24	63	2
Forage switch	Reward	Act.	Precentral gyrus	(-40, -24, 64)	3.85	8	3
Forage switch	Pre-disc. reward plus post-disc. reward	Act.	Striatum	(-16, 10, 0)	4.51	199	3
Forage switch	Pre-disc. reward plus post-disc. reward	Act.	Precentral gyrus	(-40, -22, 64)	3.93	10	5

Note. All Z > 3.1, *P* < 0.0001. All time-locked actions are from the pre-discovery phase unless otherwise specified.
*Where the cluster activation/deactivation overlaps with the ROI.

Table S6.

Two-Way ANOVA Testing the Effects of Cortical ROI and Sub-cortical ROI on the Extent to which Functional Connectivity is Moderated by Switching to Check (Related to Fig. 4Ai)

	sum_sq	df	F	PR(>F)
C(Cortical ROI)	0.024722	1.0	7.032443	0.008719
C(Sub-cortical ROI)	0.004118	1.0	1.171459	0.280549
C(Cortical ROI):C(Sub-cortical ROI)	0.056697	1.0	16.128202	0.000087
Residual	0.632770	180.0	NaN	NaN

Note. Cortical areas included ACC and AI and subcortical areas included Hb and DRN. Data entered into the ANOVA were both early and late peaks selected (using a leave-one-out procedure on the group signal) from parameters fit to β_6 from the following regression:

$$Subcortical\ ROI\ time\ course\ (Check\ switch\ and\ forage\ switch, pre\ disc.\ phase \sim \beta_0 + \beta_1 SwitchToCheck + \beta_2 TimePressure + \beta_3 Time + \beta_4 Reward + \beta_5 + CorticalROI + \beta_6 (CorticalROI * SwitchToCheck) + \beta_7 (CorticalROI * TimePressure) + \beta_8 (SwitchToCheck * TimePressure) + \beta_9 (CorticalROI * SwitchToCheck * TimePressure)$$

Table S7.

Post-Hoc Tukey HSD Test To Examine the Main Effect of Cortical ROI in ANOVA (Related to Fig. 4Ai)

Group1	Group2	Meandiff	p-adj	Lower	Upper	Reject
ACC	AI	-0.0232	0.0117	-0.0411	-0.0052	True

Table S8.

Two-Way ANOVA Testing the Effects of Cortical ROI and Sub-cortical ROI on the Extent to which Functional Connectivity is Moderated by Switching to Check and Threat Level (Related to Fig. 4Bi)

	sum_sq	df	F	PR(>F)
C(Cortical ROI)	0.023501	1.0	4.229004	0.041182
C(Sub-cortical ROI)	0.061755	1.0	11.113015	0.001041
C(Cortical ROI):C(Sub-cortical ROI)	0.004722	1.0	0.849757	0.357855
Residual	1.000259	180.0	NaN	NaN

Note. Cortical areas included ACC and AI and subcortical areas included Hb and DRN. Data entered into the ANOVA were both early and late peaks selected (using a leave-one-out procedure on the group signal) from parameters fit to β_9 from the following regression:

$$Subcortical\ ROI\ time\ course\ (First\ checks\ and\ first\ forages, pre\ disc.phase) \sim \beta_0 + \beta_1 * SwitchToCheck + \beta_2 * TimePressure + \beta_3 * Time + \beta_4 * Reward + \beta_5 + CorticalROI + \beta_6 * (CorticalROI * SwitchToCheck) + \beta_7 * (CorticalROI * TimePressure) + \beta_8 * (SwitchToCheck * TimePressure) + \beta_9 * (CorticalROI * SwitchToCheck * TimePressure)$$

Table S9.

Post-Hoc Tukey HSD Test to Examine the Main Effect of Sub-Cortical ROI in ANOVA (Related to Fig. 4Bi)

Group1	Group2	Meandiff	p-adj	Lower	Upper	Reject
DRN	HB	-0.0366	0.0011	-0.0585	-0.0148	True

Table S10.

Post-Hoc Tukey HSD Test to Examine the Main Effect of Cortical ROI in ANOVA (Related to Fig. 4Bi)

Group1	Group2	Meandiff	p-adj	Lower	Upper	Reject
ACC	AI	0.0226	0.0467	0.0003	0.0449	True

Table S11.

PPI Results: Two-Sided Wilcoxon Signed Rank Tests

ROI	Seed ROI	Regressor[2]	*P* value	Z	Mean	SD	Mean peak time[1] (s)
Hb	ACC	ACC*Check	0.001	3.28	0.05	0.06	-0.88
DRN	AI	AI*Threat*Check	0.048	1.98	0.02	0.07	4.49
DRN	AI	AI*Threat*Check	0.024	2.25	0.04	0.07	4.98

SN	Hb	Hb*Threat*Check (Successful outcome)	0.015	-2.43	-0.10	0.20	8.61
VTA	Hb	Hb*Threat*Check (Successful outcome)	0.003	-2.95	-0.10	0.13	8.71
DRN	Hb	Hb*Threat*Check (Successful outcome)	0.036	-2.10	-0.08	0.19	7.43
Hb	AI	AI*Threat	0.031	2.16	0.04	0.08	2.42
SN	Hb	Hb*Threat*Check	0.031	2.16	0.03	0.06	6.35
VTA	Hb	Hb*Threat*Check	0.033	2.13	0.02	0.06	5.76
DRN	Hb	Hb*Threat*Check	0.042	2.04	0.02	0.05	5.27
Striatum	Precentral gyrus	Precentral gyrus*Reward*Forage	0.048	1.98	0.03	0.06	0.06

[1]Average time of peaks across which two-sided Wilcoxon signed rank test was significant, relative to button press onset.

Supplementary Datasets

Dataset 1.

Post-Block Mood Rating Responses (Rating Scale 0-100).
See attached Dataset1.csv.

Dataset 2.

ROI Statistics from Whole-Brain Analysis Extracted for Each Participant
See attached Dataset2.csv.

Dataset 3.

PPI Analyses: Two-Tailed Single Sample T-Tests Across Regression Coefficients
See attached Dataset3.csv.

Supplementary Methods

Forage. The average amount of food available varied randomly per second (range: 0-90 units). Participants could always see how much food was available. When foraging the fish dived down to obtain food (translated later into money; see Fig. 1B).

Check. Predators were hidden from participants' view unless participants pressed a button to 'check' a specific portion of the surrounding area (Fig. 1C). Predators appeared (after a random delay, 2-10.5s) at the edge of the screen and moved toward the fish's location at the screen centre. When the predator reached the centre of the screen it either caught the fish (causing the participant to lose one 'life') or, if the fish was in hiding (see below), the predator quickly exited the environment (ending one 'predator epoch'). If the current predator was undiscovered, the section being checked advanced clockwise to the next section of the environment at each key press; after the participant discovered the predator's location, successive key presses re-checked the same location. Predator types (e.g. shark) differed in speed (10s, 15s or 20s to reach screen centre) (Fig. 1E, F). Each block (see below) had only one type of predator, appearing one at a time, and participants were informed which before the block start.

Hide. Pressing the 'hide' button caused the fish to escape to a safe space where it could not be caught by the predator; a subsequent button press would return the fish to the centre (Fig. 1D).

Blocks. Each of 27 experimental blocks lasted 90s and had a different combination of 1) predator type (i.e. predator speed, three levels, see above) and 2) number of segments in which participants could check for predators (range 1-4). With fewer segments, more of the environment was visible during each check action and therefore fewer checks were required to survey the entire surrounding area. Each participant received one of three schedules, each having block and reward conditions randomly generated with the absolute value of all correlations between block variables kept below r = 0.3.

After each block, participants answered two questions about how they perceived the block: 1) "How stressful was the last round?" and 2) "How exciting was the last round?". Participants respond to each question by moving a slider to indicate a score between 0 and 100.

Timings. Each action involved a time cost: foraging took 1.5s, checking took 0.5s, hiding took 0.5s, and returning from hiding took 2s. Pressing one button inactivated all other buttons for the duration of that action's time cost.

Behavioural Analysis

We analysed participants' behaviour as a function of contextual factors. For button presses occurring before the predator had been discovered in the pre-discovery phase (pre-PD phase), we analysed the probability that each action was a check versus a forage given a combination of contextual factors: reward rate, an indication of the amount of reward available that was always visible on-screen; and *time pressure*, a measure of threat level computed as in Equation 1:

$$timePressure_i = \left(\frac{1}{minDelay + predatortraveltime_i}\right) * (i - lastFullCheck_i) \quad 1$$

where i is the current time point, *minDelay* is the minimum possible delay for all predators in this task (2.5s), *predator travel time*$_i$ is the number of seconds that the predator type at i takes to reach the centre of the screen, and *lastFullCheck*$_i$ is the time point at which the participant most recently completed checking all areas of the environment (i.e., when they could be certain that no predator was present). For button presses occurring after the predator had been discovered in the post-discovery (post-PD) phase, we analysed 1) the probability that each action was a check or not, 2) the probability that each action was a forage or not, and 3) the probability that each action was a hide or not, based on three contextual factors: first, reward rate; second, *time since last check*, a measure of how long it had been since the participant had last seen the predator, computed as in Equation 2:

$$timeSinceLastCheck_i = \frac{i - lastCheck_i}{predatortraveltime_i} \qquad 2$$

where i is the current time point, *lastCheck*$_i$ is the time point when the participant last saw the predator relative to the current time point i, and *predatortraveltime*$_i$ is again the time the current predator type takes to reach the centre of the screen at i; and, third, an additional measure of threat level, *proximity*, an estimate of the amount of time until the predator would arrive. In the post-PD phase this was computed as in Equation 3:

$$proximity_i = i - (delay_i + predatortraveltime_i) \qquad 3$$

where *delay*$_i$ is the actual delay for the current predator and i and *predator travel time*$_i$ are as in Equations 1 and 2. Note that *proximity* is coded as a negative number, so that as the predator approaches, numbers become higher (i.e. less negative). All analyses of choice data were computed as non-hierarchical Bayesian regression models using the package brms (3, 4) with bernoulli(link='logit') link function. Regressions for both pre- and post-discovery phases were formulated as in Equation 4:

$$y_a \sim \beta_0 + \beta_1 reward_a + \beta_2 * t_{1a} \ldots \qquad 4$$

where y_a is a binary variable indicating whether each action *a* in the relevant phase was the target action (either check, forage, or hide; post-PD actions analysed separately), *reward*$_a$ is the reward available at the time of action *a*, and t_{ia} are the relevant threat-related variables computed for action *a* (see Table S1 for results and exact formulations). Regressors were z-score normalized and weak priors were set (normal distribution with mean 0 and standard deviation 3). Four chains were run with 4000 iterations and adapt_delta set to 0.8. Model fit was checked using Rhat < 1.1 and the absence of divergent samples. Model fits that did not meet these criteria were re-run with increased samples and adapt_delta. After computing each model non-hierarchically for each participant, we conducted two-tailed single-sample *t* tests across participants' coefficient estimates to determine the parameter's effect on choice data. Where outliers existed, test statistics are only reported as significant if the test was also significant with outliers excluded.

We also analysed participants' inter-response times (IRTs) with respect to contextual factors such as action sequence. IRT was computed as the time in milliseconds between either the start of the block or the conclusion of the previous action (after the time cost associated with that action, when buttons were inactivated) and the next action. During analysis we found that participants often pressed buttons while they were inactivated (during the time cost of the most recent action) and we included these 'inactive' button presses in our IRT analyses. To the raw IRT values we applied a within-participant min-max transformation and across-participant outlier removal: Outlier values lower than the 25th quartile minus 1.5 times the interquartile range (IQR) and greater than the 75th quartile plus 1.5 times IQR across all participants were omitted. These analyses were computed as non-hierarchical Bayesian regressions using the package brms (3, 4) with 'shifted_lognormal' link function. Regressions were formulated as in Equation 5:

$$IRT_a \sim \beta_0 + \beta_1 reward_a + \beta_2 time_a + \beta_3 block_a + \beta_4 x_{1a} + \beta_5 x_{2a} \ldots \qquad 5$$

where $reward_a$ is the current reward rate at action a, $time_a$ is the amount of time elapsed in the experiment at action a, $block_a$ is the block index for action a, and x_{ia} are contextual variables specific to each model (e.g., behavioral switch type) for action a. Modelling settings, model fit criteria, and parameter testing methods were the same as those used for the button press analysis (above).

Whole-brain analyses. Whole-brain statistical analyses were performed at two-levels as implemented in FSL FEAT (5, 6). At the first (individual) level, we used a univariate general linear model (GLM) framework for each participant to estimate parameters. To account for temporal autocorrelations, first-level data were pre-whitened before group-level analysis (6). The contrast of parameter and variance estimates from each participant were then combined at the second (group) level in a mixed-effects analysis (FLAME 1+2). The results were cluster-corrected with the voxel inclusion threshold Z=3.1 and cluster significance threshold of p<0.001 two-tailed.

First-level analyses searched across the whole brain for voxels in which BOLD signal was associated with parametric variation in model variables. Our analysis split the time from the start of the delay period to the arrival of each predator ('predator epochs') into two phases: before the predator was discovered ('pre-predator discovery', or pre-PD phase) and after ('post-predator discovery' or post-PD phase). Model variables (Fig.S1 illustrates correlation matrix) were computed separately for each phase. In the whole-brain analyses, a single GLM was used across the whole task. In the ROI analyses (see below), pre-PD and post-PD phases were analysed using separate GLMs. Importantly, in the whole-brain analysis, beyond the regressors detailed in the equations (GLM1, GLM2) below that were time-locked to action transitions, we also controlled for all other actions (all button presses related to foraging, checking and hiding, with forages and pre-PD checks segmented into first and subsequent actions), epoch threat type indexed by predator speed (slow, medium, and fast), and predator speed at the moment of predator discovery (Fig.S1).

For the pre-PD phase (GLM1), model variables included (see behavioural regressions): *reward* and *time pressure* (see Eq. 1), formulated as in Equation 6:

$$GLM1: BOLD = \beta_0 + \beta_1 reward + \beta_2 timePressure \qquad 6$$

where *BOLD* is a column vector of time series data for a given voxel time-locked to a behavioral switch. For the post-PD phase a very similar model (GLM2) was used but now, after seeing the predator, participants had access to more information to estimate threat level and so model variables included: *reward*; *time since last check* (see Eq. 2), and *proximity* (see Eq. 3), formulated as in Equation *7*:

$$GLM2: BOLD = \beta_0 + \beta_1 reward + \beta_2 timeSinceLastCheck + \beta_3 proximity \qquad 7$$

where *BOLD* is again voxel time series data time-locked to a behavioural switch. Variables *time since last check* and *proximity* were both included because they measured distinct threat-related information (correlated at $r = 0.45$ for switches to checking, $r = 0.41$ for switches to foraging, and $r = 0.04$ for switches to hiding; see correlations in Fig.S1). Regressors were modelled as stick functions (i.e., duration of zero), convoluted with a double-gamma hemodynamic response function (HRF). To reduce noise in the BOLD signal we added several task-unrelated confound regressors, including head motion parameters estimated by MCFLIRT in pre-processing; voxel-wise regressors created by physiological noise modelling (PNM) (7) to model the effects of cardiac and respiratory noise; and regressors to remove timepoints corrupted by large motion that could not be corrected with MCFLIRT (across participants, 4±1% of timepoints were marked as corrupted by large motion).

Whole-brain analyses were time-locked to actions that represented behavioural switches (e.g., the first check after a series of forages). Time-locking to behavioural switches allowed enough time to separate events for analysis. Effect Required statistics provided by lower-level FSL FEAT (6), a measure of each contrast's efficiency/estimability, indicated that the average BOLD percent signal change required for any contrast of interest in the pre-discovery phase across all participants was 1.83 ± 0.58 (maximum 2.595). Due to participants conducting fewer checks after discovering a predator, contrasts in the post-discovery phase were more difficult to estimate; average BOLD percent signal change required across post-discovery contrasts was 2.46 ± 0.91 (maximum 3.64). Consequently, tests of post-discovery phase data (below) used only data from participants who checked >40 times and whose first-level statistics reported Effect Required below 2% for post-discovery check switch contrasts (N=13) or post-discovery forage switch contrasts (N=12).

<u>*ROI time course analyses.*</u> To study the activity of regions of interest (ROIs), anatomical masks were created for each ROI in the MNI standard space using a conversion of the Talairach structural atlas (transformed into MNI space (8–10)), mask templates from similar studies (2, 11, 12), and cluster-corrected activations identified via whole-brain analysis. Next, masks were transformed from standard space to each participant's structural space by applying a standard-to-structural warp field, transformed from structural to functional space by applying a structural-to-functional affine matrix, and binarised.

These masks were used to extract time-series data for analysis. First, a first-level whole-brain analysis was conducted for each participant with only regressors of no interest (all forages and all pre-discovery checks). Time-series data from each voxel within each ROI were then extracted from the residual functional data. Next, time-series data were averaged across the voxels within each ROI, normalised, up-

sampled 20 times with cubic spline interpolation, and epoched in 17s windows starting from 2s before the button press to 15s after. Finally, GLMs were fit to each time step of the epoched data.

We partitioned the ROI time courses into two phases for analysis: an early phase (0-5s post-action) when neural activity associated with the action of interest first becomes observable, and a late phase (5-10s post-action) that may reflect secondary neural processes associated with the action of interest. For each psychophysiological interaction (PPI) analysis, time courses were time-locked to action switches in the pre-discovery phase and regressions were formulated as in Equation 8:

$$\begin{aligned} BOLD \sim \beta_0 &+ \beta_1 switch_i + \beta_2 time_i + \beta_3 reward_i + \beta_4 timePressure_i \\ &+ \beta_5 ROI2_i + \beta_6 (ROI2_i * switch_i) + \beta_7 (ROI2_i * v_i) \\ &+ \beta_8 (switch_i * v_i) + \beta_9 (ROI2_i * switch_i * v_i) \end{aligned} \quad (8)$$

where *BOLD* is a *i* x *t* (*i* button press action, *t* time samples) matrix containing the time-series data from a given ROI during both switching to check and switching to forage; $switch_i$ is a binary variable indicating the behavioural switch (switch to check or forage) associated with action *i*, with the switch of interest coded positively; $time_i$ is the time elapsed in the experiment at action *i*; $reward_i$ is the reward rate available at the time of action *i*; *time pressure*$_i$ is a measure of threat level at action *i* (see Eq. 1); *ROI2* is the time course for another area of interest at action *i*; and v_i is either *reward* or *time pressure* from action *i* depending on the effect of interest. To test for significance, we searched for peaks (or troughs) in each phase (early and late; see above) using a leave-one-out procedure to avoid any temporal selection biases: for a parameter of interest, a β weight value was selected for each participant from the time point identified as the peak average signal for the group minus that participant (similar to the approach used in Khalighinejad et al (2, 11). Selected values were tested via two-tailed single sample t tests. Further correction for multiple comparisons was considered unnecessary because ROIs were chosen based on their significance in cluster-corrected whole-brain analysis (Z>3.1; p<0.001 two-tailed), which itself performs rigorous correction for multiple comparisons.

To test for overall effects of factors such as ROI location (cortical versus sub-cortical) and behavioural switch type on functional connectivity, we performed ANOVAs on fitted parameter peak values selected using the same method. Peaks from both the early time window and late time window were included in ANOVAs.

*<u>Post-PD phase analyses</u>***.** All the previous analyses focused on activity in distributed neural circuits linked to decisions to forage for rewards or check for threats in the pre-PD task phase. We initially focused on this task phase because it contained similar levels of checks and forages. In the final stage of the analysis, however, we examined whether we could reproduce findings from the pre-PD phase by conducting similar analyses in the post-PD phase. In this way it was possible to perform a series replication tests albeit within the same participants rather than an independent sample. It was not possible to analyse the data for all participants in the post-PD phase because some only made a very small number of checks during this task phase. When testing the possibility that we could find similar results, we therefore focused on participants who had made more checks (>40) in the post-PD phase and in whom an analysis of effects sizes indicated <2% change in BOLD signal was required to detect an effect of the parameter of interest (threat level or reward) during switches to check (leaving N=12 in all post-PD phase analyses). We took an analogous

approach when testing the replicability of the results in Figure 6 albeit now focusing on participants in whom an analysis of effects sizes indicated <2% change in BOLD signal was required to detect an effect of the parameter of interest during switches to forage (leaving N=12 for within-subject replication analyses related to foraging and N=13 for those related to checking).

To test whether key findings from our fMRI analysis replicated, we regressed variables of interest against ROI time course data with models formulated as in Equation 9:

$$ROI \sim \beta_0 + threat_i + \beta_2 reward_i + \beta_3 time_i \tag{9}$$

where *ROI* is the time course data processed as in the PPI analyses; $threat_i$ is a measure of threat level at action *I* as indexed by either *time pressure* or *proximity* for the pre- and post-discovery phases respectively; $reward_i$ is the reward available at action *I*; and $time_i$ is the time elapsed at action *i*. Models were fit for each ROI in the distributed neural circuits presented in the main text (ACC, AI, DRN, Hb, SN, and VTA), with separate models for the pre- and post-discovery phase data. Peaks were selected from parameters fit to pre-discovery phase data using the leave-one-out procedure (above). Here the peak search was constrained to a time window dictated by significant PPI results reported in Table S11: For tests involving ROIs in the threat-related circuit, we used the range of mean peak times reported for all PPIs associated with action commission within that circuit. Instead of searching for PPI effects occurring at the exact same time as the pre-PD phase, we used this peak search procedure to slightly expand the search window to allow for task-related differences between the pre- and post-PD phases: due to fewer repeated checks in the post-PD phase, the timing of behavioural switch effects might be expected to be slightly different. Selected peaks were tested with two-tailed single sample t-tests. Tests significant at the $p < 0.05$ level were then conducted using the equivalent parameters fit to post-PD phase data. Peaks were selected from these parameters by searching within the time range that contained 95% of the selected peaks from the equivalent pre-PD phase test. Selected peaks were then tested with a one-tailed single-sample t-test, with the tail corresponding to the direction of effect in the pre-PD phase test.

References

1. O. Hikosaka, The habenula: From stress evasion to value-based decision-making. *Nat Rev Neurosci* **11**, 503–13 (2010).
2. N. Khalighinejad, L. Priestley, S. Jbabdi, M. F. S. Rushworth, Human decisions about when to act originate within a basal forebrain-nigral circuit. *Proc Natl Acad Sci USA* **117**, 11799–11810 (2020).
3. P. C. Bürkner, brms: An R package for Bayesian multilevel models using Stan. *J Stat Softw* **80**, 1-28 (2017). doi.org/10.18637/jss.v080.i01
4. P. C. Bürkner, Advanced Bayesian multilevel modeling with the R package brms. *The R Journal* **10**, 395-411 (2018). doi.org/10.32614/RJ-2018-017
5. M. W. Woolrich, T. E. Behrens, C. F. Beckmann, M. Jenkinson, S. M. Smith, Multilevel linear modelling for FMRI group analysis using Bayesian inference. *Neuroimage* **21**, 1732–1747 (2004).
6. M. W. Woolrich, B. D. Ripley, M. Brady, S. M. Smith, Temporal autocorrelation in univariate linear modeling of FMRI data. Neuroimage **14**, 1370–1386 (2001).

7. J. C. W. Brooks *et al.*, Physiological noise modelling for spinal functional magnetic resonance imaging studies. *Neuroimage* **39**, 680–692 (2008).
8. J. L. Lancaster *et al.*, Bias between MNI and Talairach coordinates analyzed using the ICBM-152 brain template. *Hum. Brain Mapp.* **28**, 1194–1205 (2007).
9. J. Talairach, P. Tournoux, Co-Planar Stereotaxic Atlas of the Human Brain (Thieme, 1988).
10. J. L. Lancaster *et al.*, Automated Talairach atlas labels for functional brain mapping. *Hum. Brain Mapp.* **10**, 120–131 (2000).
11. N. Khalighinejad, N. Garrett, L. Priestley, P. Lockwood, M. F. S. Rushworth, A habenula-insular circuit encodes the willingness to act. *Nat. Commun.* **12**, 6329 (2021).
12. M. C. Klein-Flügge *et al.*, Relationship between nuclei-specific amygdala connectivity and mental health dimensions in humans. *Nat. Hum. Behav.* **6**, 1705–1722 (2022).
